# A spatially differentiated water pollution policy leads to economic and health inequity

**DOI:** 10.1073/pnas.2421404122

**Published:** 2025-09-24

**Authors:** Zhonghui Luo, Lala Ma, Rui Xie, Ran Song

**Affiliations:** ^a^School of Economics and Trade, Hunan University, Changsha 410006, China; ^b^Graduate School of Economics, Kyoto University, Kyoto 606-8501, Japan; ^c^Department of Economics, University of Kentucky, Lexington, KY 40506; ^d^Resources for the Future, Washington, DC 20036; ^e^Department of Economics, National University of Singapore, Singapore 117570, Singapore

**Keywords:** environmental justice, firm behaviors, health inequity, economic inequity

## Abstract

Disparities in air pollution exposure by socioeconomic status (SES) are well documented. However, there is much less evidence on the inequitable consequences of water pollution and policies affecting water pollution gaps. This study uncovers the distributional impact of China’s 11th Five-Year Plan (2006–2010), which triggered spatially differentiated water pollution abatement for a population of almost 1.3 billion. We document that the policy reduced water pollution overall, targeting areas based on pollution level. Since high pollution areas tend to be wealthier, this policy concentrated firm water pollution abatement and reductions in polluting firm entry in higher-SES areas. While pollution fell, the policy further widened the gap in health and economic outcomes, particularly for those without tap water access: Disparities in risks of tumor, cardiovascular disease, labor supply, and wages increased. Our findings demonstrate that policy-induced changes in water pollution can interact with unequal access to defensive infrastructure to exacerbate SES gaps in outcomes. The analysis also suggests that consideration of heterogeneous pollution vulnerability across the population may affect the overall benefits of a policy.

## Significance

Little is known about how environmental policies alter water pollution gaps and thereby affect well-being inequities. We study China’s 11th Five-Year Plan, which triggered spatially differentiated water pollution abatement and targeted high pollution areas. Because more polluted areas coincided with higher-socioeconomic status (SES) areas, the policy led to greater reductions in firm emissions and polluter entry in higher- vs. lower-SES areas. The policy thus improved water quality for all and narrowed exposure gaps by SES. However, these changes exacerbated economic and health inequity in areas with poor drinking water infrastructure: Disparities in risks of tumor, cardiovascular disease, and labor supply increased in these communities. Our analysis highlights the role of defensive infrastructure in shaping the distributional effects of environmental regulation.

Environmental pollution is a major health risk factor, undermining human well-being and economic prosperity. It is well documented that disparities in air pollution exposure exist both across regions and by social status (1, 2). However, little is known about the distributional consequences of water pollution and policies affecting water pollution gaps. With increasing evidence that people with low socioeconomic status (SES) are considerably more vulnerable to pollution, a question arises about the extent to which water pollution is a contributor to health and economic inequity and how regulations have impacted gaps in water pollution exposure.

**Fig. 1.**
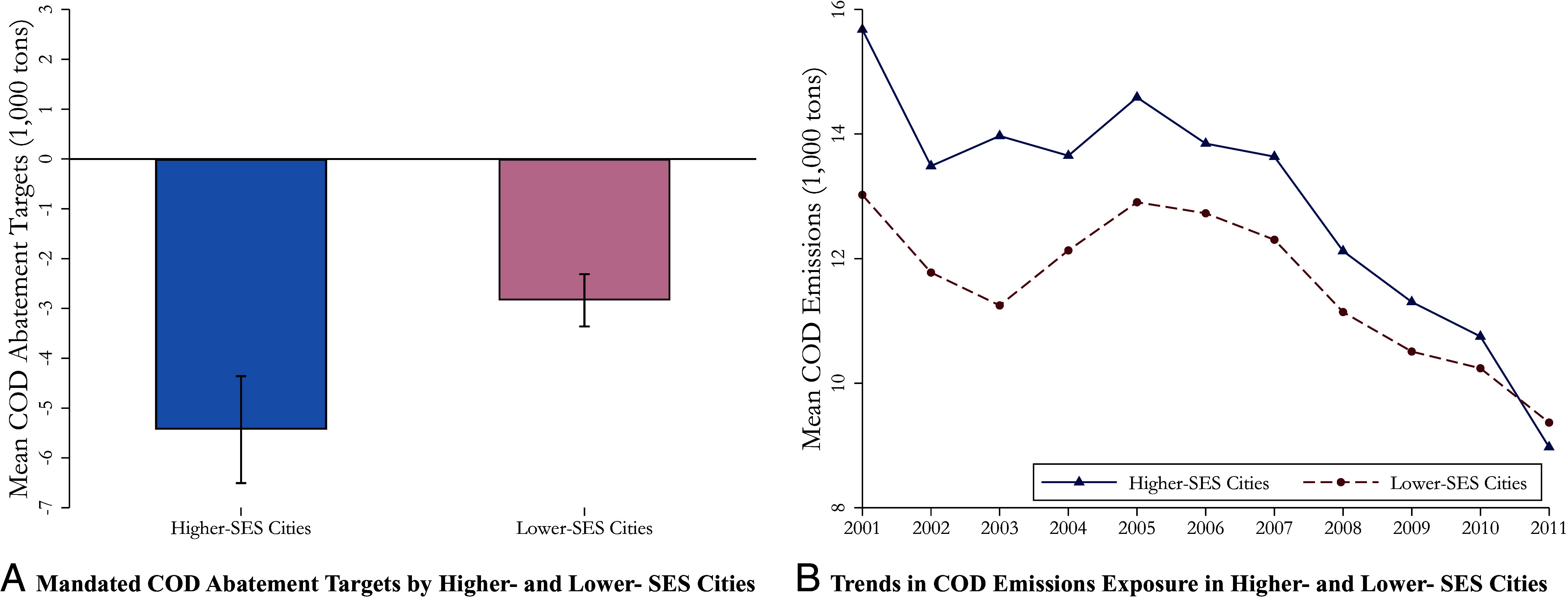
COD Abatement Targets and Trends in COD Emissions Exposure. Notes: We aggregate firm-level COD emissions to the city-by-year level and divide cities into two groups based on whether the baseline fraction of unskilled people in 2000 is above the national median. Skill-level is assigned based on whether an individual has a college degree. In panel (*A*), we show average COD abatement targets for higher- and lower- SES cities, respectively, and include 95% CIs. In panel (*B*) we show average city-level COD emissions in each of the two groups (lower-SES cities and higher-SES cities) from 2000 to 2011.

This paper uses China as a laboratory to study the distributional consequences of changes in Chinese firms’ water pollution activity as a result of China’s 11th Five Year Plan (FYP) (2006–2010). Since 2006, China invested over 82 billion Chinese Yuan in water pollution abatement and introduced spatially differentiated water pollution abatement targets through the 11th FYP (3). During 2006–2010, China’s total Chemical Oxygen Demand (COD) emissions decreased overall (*SI Appendix*, Fig. S1) as the policy targeted high pollution areas across the nation. Since the most polluted areas in China coincide with cities where individuals are economically better off, the COD reduction targets were considerably more stringent in rich relative to poor areas (Fig. 1*A*). As illustrated in Fig. 1*B*, water pollution was initially greater in higher-SES areas before the policy. Beginning in 2006, average COD emissions began falling for all communities, but the decrease was larger for higher- relative to lower-SES areas, effectively making the distribution of pollution more equal, but less progressive. By 2011, the gap in average COD emissions between lower- and higher- SES cities narrowed to nearly zero.

Our study investigates whether the pattern of pollution abatement in the 11th FYP risks generating unintended inequitable consequences on well-being, given persistent unequal access to defensive infrastructure. Environmental policies that mitigate pollution based on pollution levels are rational from a cost–benefit perspective. In many cases, there is an inherent trade-off between the goals of efficiency and equity in policymaking, which extends to the environmental policy setting (4). In China, because high pollution areas coincide with higher-SES areas, a policy targeting pollution levels is likely to trigger less abatement for low-SES communities. Indeed, in our context, the 11th FYP effectively reduced pollution levels for all, but differentially so in high pollution areas where high-SES individuals reside. Since all groups experienced pollution reduction, this policy likely improved overall welfare compared to a counterfactual scenario without any pollution reduction. However, what matters most is not exposure in and of itself, but the damages resulting from that exposure. When there are large differences in vulnerability across populations such that low-SES individuals are more vulnerable (due to, e.g., the ability to undertake avoidance measures and defensive expenditures), then the marginal damage from pollution is likely greater for the low-SES group and targeting greater pollution reductions in higher-SES areas may not only increase disparities in well-being but also be inefficient. In other words, a more equal allocation of pollution abatement across SES, all else constant, may improve health protection and benefits overall because changes in pollution exposure may not translate into the same change in health outcomes due to unequal vulnerabilities.

Using administrative records on COD emissions for over 127,000 firms and firm-level establishment data over time, we estimate how China’s 11th FYP altered the distribution of water pollution in China using a difference-in-differences (DD) framework. We first characterize an area’s SES based on demographic characteristics in the baseline year of 2000. We then compare changes in firm emissions after the policy was enacted in 2006 between firms located in areas with higher vs. lower concentrations of low-SES individuals, uncovering the change in the level and the gap in water pollution emissions by SES. Specifically, our analysis focuses on within-firm changes in water pollution behavior that removes spatial trends and firm-level unobservables through province-by-year and firm fixed effects. Additionally, we examine heterogeneity in the stringency of water pollution targets by SES to provide further support that the changes in water pollution activity that we detect are linked to water pollution targets established by the FYP.

While the pollution control policy reduced exposure gaps, access to defensive infrastructure, i.e. tap water, remained much lower for low-SES individuals, with the disparity persisting throughout the 11th FYP (*SI Appendix*, Table S1). Given the potential for differential vulnerability to pollution, we next investigate the policy’s impact on gaps in health, labor supply, and wages between high and low SES groups. We employ longitudinal survey data at the individual level and use a triple-differences framework to examine how health and labor outcomes changed following the policy, comparing individuals across skill levels and by access to tap water. This design allows us to identify how the policy’s effects on disparities in well-being vary with access to defensive infrastructure. Lower mandated COD reductions for lower-SES areas combined with persistently limited access to purified water for low-SES individuals may ultimately translate into increased health inequities.

We document that China’s 11th FYP triggered spatially differentiated water pollution abatement. Since abatement quotas were significantly less stringent in lower-SES areas, the magnitude of firm-level COD emission reductions was smaller for areas with higher shares of low-SES individuals. Moreover, the 11th FYP led to smaller reductions in the entry of polluting firms in lower-SES areas. This pattern is confirmed using a host of fixed effects to remove unobserved confounders that control for time-invariant, firm-level unobservables and differential industry trends that may affect abatement decisions. Estimates are robust when controlling for other water control policies enacted around the same time period and are not driven by other major policy goals under the 11th FYP or changes in air pollution (SO_2_).

Using individual-level longitudinal panel data, we find that the policy widened disparities in health and labor market outcomes, despite overall improvements in water quality. In particular, among individuals without access to tap water, the policy led to increased gaps between unskilled and skilled individuals in the incidence of tumors and cardiovascular diseases, as well as in daily work hours and wages. The effects are not found for those who have access to tap water and for illnesses that are unlikely related to water pollution (e.g., asthma and respiratory diseases). Our results remain robust to accounting for access to health and medical services, age-related variation in health outcomes, and other contemporaneous water pollution policies. We also perform placebo tests using access to an indoor bathroom (instead of tap water)-a factor that may influence health through channels unrelated to water pollution. Our pattern of health gaps disappears in these placebo tests.

To summarize, the 11th FYP improved water quality for all individuals, but generated differential improvements across different SES groups. Higher-SES areas, which initially faced higher levels of water pollution, experienced greater pollution abatement. Our paper highlights that low-skilled individuals had more limited access to purified tap water than the high skilled throughout the entire period of the 11th FYP. The resulting differences in vulnerability are likely amplified when additionally considering private measures of avoidance behavior. The combination of spatially uneven pollution reduction and persistent unequal access to defensive infrastructure widened disparities in health and labor market outcomes. We posit that a policy to increase investment in defensive infrastructure, particularly for low-SES communities, may improve social welfare and reduce well-being inequities. However, we caveat that policy evaluation requires accounting for implementation and other costs, as well as any general equilibrium price effects. Therefore, understanding the full welfare implications of alternative policies would require accounting for all of the costs and benefits, which is beyond the scope of our analysis.

We aim to provide the following contributions to the literature. First, our work fits in the recent literature on environmental justice (1, 5). Government interventions and firm strategic behavior may impact gaps in air pollution exposure (6–9). There is a lack of studies directly identifying firm behaviors under environmental regulations and consequent changes in water pollution by SES. We show how environmental regulations can alter exposure gaps by SES in the context of water pollution using firm-level evidence and highlight spatially differentiated government action as causal mechanism underlying environmental justice correlations(1, 10).

Second, we emphasize the role of defensive infrastructure in reshaping gaps in individual health and labor market outcomes as a result of changes in the distribution of pollution exposure. Low SES individuals are considerably more vulnerable to pollution due to limited resources and higher avoidance costs (11–13). Our analysis demonstrates that unequal access to defensive infrastructure exacerbates this vulnerability in China and can interact with pollution control policies to widen disparities in health and economic outcomes. Our findings propose environmental health and gaps in pollution vulnerability due to defensive infrastructure as potential drivers of enduring within-country gaps in welfare documented in the literature (14–16). Whether differential vulnerability ultimately translates into gaps in well-being is essential to understanding the welfare and distributional impacts of environmental policy.

Third, we speak to the literature on water pollution in China (17–24). Work across various disciplines has documented infant mortality and high incidences of water-borne and carcinogenic diseases in China associated with high levels of water pollution (25–27). This literature highlights the insufficiency of basic water supply and sewage treatment infrastructure in China that occurs predominantly in rural areas, which is hypothesized to partially explain disparities in health outcomes by socioeconomic status (20, 21, 23). Additionally, literature in economics has examined how water pollution responds to regulatory incentives in China (18), consistent with the formation of domestic pollution havens (28–30). We build on these notable works by documenting how China’s spatially differentiated water pollution regulation changed the exposure gap by SES through impacting firm pollution behavior, and how the policy’s enforcement efforts combined with differential access to health-protective measures exacerbated well-being inequality in China.

## Background

In the 11th FYP for 2006–2010, China’s central government established environmental quality as a clear policy objective. Specifically, the 11th FYP adopted environmental protection goals to reduce COD emissions by 10%^*^ or a total of 1.41 million tons from their 2005 levels (31).^†^ City-specific COD abatement mandates in the 11th FYP significantly lowered firm-level COD emissions, and the effects are robust to accounting for industry by year level fixed effects (*SI Appendix*, Table S2).

This regulation’s intensity varies across space. The Ministry of Environmental Protection (MEP) and the National Development and Reform Committee (NDRC) of China published *the 11th Five-Year National Total Emissions Control Plan of the Major Pollutants* to specify and allocate reduction targets at the provincial level (32). Coastal provinces, where pollution levels were higher, were subject to significantly higher reduction mandates than inland provinces. Notably, since coastal areas coincide with richer provinces, this meant that reduction mandates were less stringent for poorer, inland provinces, where the unskilled and poor are concentrated. The distribution of pollution targets is also uneven within each province. In November 2006, the MEP issued a within-province allocation guideline, indicating how much each city must reduce its COD (32). The within-province allocation of COD reduction target was based on a city’s contribution to the total COD emissions at the provincial level in 2005. As the amount of emissions was much higher in rich and early industrialized cities before China’s 11th FYP, the guideline allocated stronger emission abatement mandates to developed cities, but moderate mandates to underdeveloped cities within the province. Compared to residents in rich cities, those in underdeveloped cities tend to be poorer and less educated. We confirm that the stringency of water pollution mandates indeed varies with sociodemographics, where mean COD reduction targets for cities with higher (than median) shares of disadvantaged groups in terms of education, rurality, and occupation see significantly lower mandated COD reductions (*SI Appendix* Table S3).

*SI Appendix*, Table S4 summarizes the average COD emissions for two city groups (defined based on the median baseline share of unskilled individuals) before and after the implementation of the policy. Before the policy, the average COD emissions were 14.3 thousand tons in higher-SES cities and 12.2 thousand tons in lower-SES cities. During the 11th FYP, COD emissions in higher-SES cities decreased by up to 1.9 thousand tons, while lower-SES cities experienced a more modest reduction of 0.8 thousand tons.

Disadvantaged groups, who experienced smaller reductions in pollution exposure, are persistently more vulnerable to water contamination (*SI Appendix*, Table S1): In 2004, the year before the FYP, the share of people with tap water was between 25 and 30 percentage points (pp) higher for more educated individuals (high school or college degree) and this remained around 25 to 26 pp in 2009.^‡^ While China’s pollution regulations in the 11th FYP lowered overall levels of COD emissions, the pattern of spatially differentiated pollution abatement mandates reduced pollution disproportionately in places with higher concentrations of advantaged groups who have more health-protective measures against water pollution.

We next describe the trends reflected in the raw data. *SI Appendix*, Fig. S2*A* replicates Fig. 1*B*, showing that both lower-SES and higher-SES cities experienced a decline in average COD emissions, with a substantially larger reduction observed in higher-SES cities. *SI Appendix*, Fig. S2*B* illustrates the differences in emission exposure between the two groups of cities, conditional on provincial FEs. The emissions gap was substantial and significantly negative prior to 2006, became smaller in magnitude and statistically insignificant following the launch of the 11th FYP (i.e., in 2006), and eventually narrowed to nearly zero by 2011. *SI Appendix*, Fig. S2*C* shows the raw trends in cardiovascular illness. Following the implementation of the policy, disparities in the incidence of cardiovascular illness by SES widened substantially. *SI Appendix*, Fig. S2*D* illustrates that daily work hours among high-SES individuals remained stable over time, while those in the low-SES group experienced a decline in working hours after the policy was enacted.^§^

## Results

### Results on Inequities in Firm-level Activity.

#### Baseline results on firm activity.

Table 1 presents our estimates of how the policy differentially affects emissions based on an area’s SES. Table 1 column 1 presents the results of a baseline difference-in-differences regression. We regress city-level COD emissions on a continuous measure of low-SES status (i.e. share of individuals without high school or college degrees), a post-2005 dummy, and their interaction. As reported in Panel (*A*), the coefficient on the share of below-college populations is negative, though not statistically significant at conventional levels. This suggests that a one-standard-deviation (SD) increase in the percentage of below-college populations (4.7 p.p) is associated with a decrease of 0.99 thousand tons (4.7 × 0.21) of city-level COD emissions during the preperiod. Panel (*C*) of Table 1 provides summary statistics of key variables of interest and shows that average city-level emissions decreased by 1.4 thousand tons following the policy (13.27 to 11.87).^¶^ Our primary variable of interest—the interaction between the share of below-college population and the post-2005 indicator—is significantly positive, while the coefficient on the post-2005 indicator is significantly negative.^#^ This implies that all cities experienced some level of pollution abatement, with the extent varying according to the SES composition of their populations. A city with a one-SD larger baseline share of below-college populations (4.7 p.p) would experience 0.78 thousand tons (0.165 × 4.7) less emission abatement, which corresponds to 6% of mean COD emissions in the preperiod. Column 2 controls for city and province-by-year fixed effects to account for city-level time-invariant confounders and China’s various policies on environmental protection, public welfare, and industrial development, which likely varied largely by province. Inclusion of these controls increases the magnitude and significance of the coefficient estimates of the interaction between the below-college population share and the post-policy indicator.

**Table 1.** Impact on emission behaviors by socioeconomic status

	(1)	(2)	(3)	(4)	(5)
	City-level		Firm-level	City-level	
Dep. var.:	COD emission (1,000 tons)		COD emission (ton)	Firm entry	Firm exit
*Panel A: Population share without college education*					
Share of below college × Post_05_	0.165***	0.292**	0.358***	0.623**	0.280
	(0.026)	(0.119)	(0.106)	(0.266)	(0.487)
Share of below college	−0.208				
	(0.141)				
Post_05_	−16.426***				
	(2.873)				
*Panel B: Population share without high school education*					
Share of below HS × Post_05_	0.099***	0.218**	0.232**	0.453**	0.089
	(0.005)	(0.090)	(0.079)	(0.195)	(0.333)
Share of below HS	−0.213*				
	(0.100)				
Post_05_	−9.465***				
	(0.564)				
Observations	3,327	3,327	529,671	3,000	2,998
Fixed effects (Applies to panels A & B):					
Province FE	X				
City FE		X		X	X
Firm FE			X		
Province × Year FE		X	X	X	X
*Panel C: Summary statistics of key variables*					
Preperiod mean of dep. var.	13.269	13.269	34.803	23.170	31.999
Post-period mean of dep. var.	11.868	11.868	32.133	9.722	40.532
Mean of share of below college	91.021	91.021	91.021	91.021	91.021
SD of share of below college	4.746	4.746	4.746	4.746	4.746
Mean of share of below HS	81.644	81.644	81.644	81.644	81.644
SD of share of below HS	7.699	7.699	7.699	7.699	7.699

*Notes:* Share of below high school denotes the percent of the population without a high school degree. Share of below college denotes the percent of the population without a college degree. Column 1 additionally controls for city tiers. Two-way robust SEs clustered at province and year levels are reported in parentheses.

***P< 0.01, **P< 0.05, *P< 0.1

Changes in city-level emissions are driven by how polluting firms responded to the policy. Column 3 of Table 1 Panel (*A*) further examines the effect on firm-level emissions. We additionally add firm fixed effects to remove unobserved differences across firms. The *Bottom* panel of Table 1 shows that average firm-level emissions declined following the policy.^‖^ Moreover, the coefficient on the interaction between the post-2005 indicator and the population share of low-SES individuals is significantly positive, indicating that cities with a higher proportion of low-SES residents experienced smaller reductions in firm emissions. Specifically, a one-SD increase in the share of individuals without a college degree is associated with 1.7 tons (4.7 × 0.36) less emission abatement (or 4.9% relative to average prepolicy firm emissions levels). Columns 4 and 5 assess firm entry and exit dynamics. According to Panel (*C*) of Table 1, cities experienced, on average, a decline in firm entry and an increase in firm exit after the 11th FYP was enacted. However, cities with a lower concentration of low-SES individuals saw a significantly smaller reduction in firm entry [Panel (*A*) column 4]. In contrast, changes in firm exits do not appear to be related to the share of low-SES populations [Panel (*A*) column 5]. Table 1 Panel (*B*) repeats our analysis using the baseline population share of individuals without a high school degree as the SES measure. We observe a similar empirical pattern-cities with a higher concentration of unskilled individuals saw lower abatement of emissions and a smaller decrease in firm entry.^**^

**Fig. 2.**
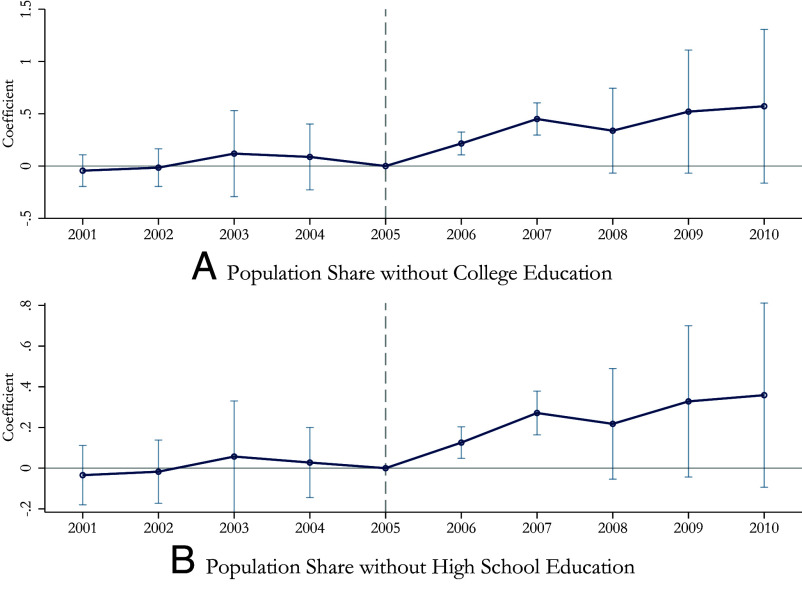
Firm Behavior Event Study (DD), Higher vs. Lower SES Areas. Notes: We test for preexisting trends and dynamics of the policy in an event study framework. The baseline year of comparison is 2005 (the year before the 11th FYP), and the x-axis shows the year relative to that baseline year. Panel A measures a city’s baseline SES by the share of individuals without a college education, and Panel B uses the share without a high school education. Vertical bands represent 95% CIs. Data come from the Environmental Survey and Reporting (ESR) and the Population Census of China.

The identifying assumption of our difference-in-differences analysis is that, in the absence of the water pollution control policy, firm COD emissions in cities with different baseline population shares of low-SES individuals would have followed parallel trends. A concern, therefore, is that our estimated effects on changes in emissions may be driven by unobserved differences between higher- and lower- SES areas that are correlated with the enactment of the 11th FYP. To examine this concern, we perform pretrends tests. Fig. 2 presents an event study of the treatment effect based on the baseline share of population without a college degree or a high school degree.^††^ We find no systematic evidence of differential pretrends in emissions across areas with different baseline SES.^‡‡^

#### Sensitivity analysis of firm activity.

##### The effect on SO2 emissions.

China’s 11th FYP also introduced spatially differentiated SO2 abatement mandates. Nevertheless, the policy’s differential effect on firm-level SO_2_ emissions across areas of lower and higher disadvantage is not statistically significant (*SI Appendix*, Table S6). Since Chinese SO_2_ abatement policy, e.g., the “Two Control Zones” Policy, was implemented much earlier than the 11th FYP (beginning in 1998) and targeted many coastal (i.e., rich) provinces, the earlier air pollution regulation is likely to have reduced the air pollution gap between rich and poor areas prior to the 11th FYP.

##### Other water pollution policies.

Three other major policies during the same period may confound our estimated effects on firm emissions. First, in April 2008, the MEP released *The Water Pollution Prevention and Control Plan for Major River Basins (2006–2010)*, which aimed to control water pollution in major waters including Huaihe River, Haihe River, Liaohe River, the mid and upper reaches of the Yellow River, Dianchi Lake, and Chaohu Lake. We create indicators for cities in protected areas (cities through which these rivers/lakes flow) and control for interactions between these indicators and year fixed effects in Panel (*A*) of *SI Appendix*, Table S7. Accounting for the confounding effects of the plan does not change the empirical patterns that we find.

Second, China introduced The River Chief Policy (RCP) beginning in 2007, which covered several provinces. The leading local officials are appointed as river chiefs for a particular river course and are made responsible for water protection and management within each of their jurisdictions. *SI Appendix*, Table S7 Panel (*B*) accounts for the effects of the RCP by controlling for an indicator for whether a city had implemented the RCP in a particular year. Our results on firm activity remain similar.

Third, since 2007, the MEP released *The List of National Key Monitoring Firms* in each year.^§§^ We manually collect the list of *National Key Monitoring Firms* for each year and add an indicator for whether a firm belonged to this list in a particular year in our firm-level regressions. We also control for a city’s total number of *National Key Monitoring Firms* when looking at firm entry and exit at the city level. *SI Appendix*, Table S7 Panel (*C*) shows that accounting for the effects of the policy does little to affect our results.

Moreover, Panels (*D* and *E*) of *SI Appendix*, Table S7 further show that our results remain robust when excluding firms located in regions covered by *The Water Pollution Prevention and Control Plan* and *The River Chief Policy*.

##### Other policy goals in the 11th FYP.

The 11th FYP is a comprehensive policy that also includes urbanization and development programs (31). If these urbanization and development programs improve economic outcomes in underdeveloped areas but worsen water pollution, then there is a concern that these programs are negatively correlated with water policy stringency and may overstate the policy difference across low and high SES groups.

Our baseline analysis controls for province-by-year fixed effects, which accounts for interprovince differences in economic development and urbanization due to the 11th FYP. To further address this concern, we use several economic development and urbanization indicators as outcome variables, including GDP, GDP growth, urbanization rate (i.e. share of populations in nonagricultural employment), and the unemployment rate. *SI Appendix*, Table S8 shows that the interaction between the population share of low-SES individuals at baseline and the post-2005 dummy cannot predict changes in these indicators during the 11th FYP. Therefore, conditional on province-by-year FEs, the implementation of urbanization and development programs is not systematically related to the geographic distribution of low-SES population.

We also regress changes in economic development and urbanization indicators between 2005 and 2010 on city-specific COD reduction targets specified in the 11th Five-Year Plan. *SI Appendix*, Table S9 shows that the COD abatement targets assigned to each city were not related to changes in most of these indicators following the policy. However, city-level abatement target is positively correlated with changes in GDP between 2005 and 2010. If water pollution is an inevitable by-product of expanding a city’s GDP, this would lead us to underestimate the policy difference in COD abatement across low- and high-SES groups.

##### Alternative measures of SES.

We examine whether our results are robust to using alternative measures of SES. *SI Appendix*, Table S10 Panel (*A*) uses the proportion of rural *hukou* holders among the population. Panel (*B*) measures SES based on occupation, where high-SES individuals correspond to those whose occupations are public sector staff, professionals, and technicians, and low-SES individuals are the remaining population. We leverage these SES measures in the baseline year of 2000. Our results document a similar pattern -cities with a higher share of low-SES individuals experienced less pollution abatement during the 11th FYP.

*SI Appendix*, Table S11 Panel (*A*) further shows that wealthier cities experienced greater COD abatement during the 11th FYP, which is also consistent with the pattern observed in our baseline results. Panel (*B*) examines how changes in COD emissions in the aftermath of the policy were related to ethnic minority population shares. Patterns are also generally consistent with our baseline results, although we observe less increase in firm exits in high minority areas.^¶¶^ This may potentially be explained by changes in policies that aim to benefit ethnic minorities that occurred during our sample period of 2006–2010.

##### Industrial structure controls and alternative specifications.

One may expect higher- and lower-SES cities to have different industries that could have been impacted differently during the 11th FYP. To assess this concern, we control for industry-by-year fixed effects in *SI Appendix*, Table S12. Our results are qualitatively similar.

We next use a discrete measure of a city’s SES to categorize treatment. We categorize cities into five groups based on the quantiles of a city’s SES at baseline^##^ and regress COD emissions on four interaction terms—specifically, interactions between the post dummy and the second, third, fourth, and fifth quantile dummies, respectively. As the policy reduced water pollution for all cities in China, the coefficients on these interactions capture the difference in COD emissions for each group relative to cities in the bottom quantile of low-SES population shares. As shown in *SI Appendix*, Fig. S4, the coefficient estimates for the third, fourth, and fifth quantile groups are significantly positive and increase monotonically. This suggests that cities with a higher baseline share of low-SES populations experienced less COD abatement during the 11th FYP, with the degree of abatement decreasing in a monotonic pattern.

##### Multiple hypothesis testing.

In our baseline estimates reported in Table 1, we assess the effects of the policy on multiple outcome variables related to emission behaviors. When testing multiple hypotheses simultaneously, standard statistical techniques may lead to overrejection of null hypotheses (34). (In multiple hypothesis testing, as the number of hypotheses increases, the probability of falsely rejecting true null hypotheses tends to increase). We use the approach proposed by refs. 35 and 36 and estimate Romano–Wolf step-down adjusted *P*-values robust to multiple hypothesis testing. The Romano–Wolf correction uses resample methods to control the familywise error rate (FWER), which is the probability of rejecting at least one true null hypothesis in a family of hypotheses being tested.

*SI Appendix*, Table S13 reports Romano and Wolf adjusted *P*-values for coefficient estimates in Table 1 and *SI Appendix*, Table S5. In all cases except for firm entry, the coefficient estimates of our primary variables of interest—the interaction between baseline shares of individuals without a college degree and the post-2005 indicator—increase in the level of significance, with Romano and Wolf *P*-values < 1%. Our findings are thus robust to adjusting for multiple hypothesis testing.

##### The power of pretrends test.

We evaluate the power of our pretrends test based on the baseline share of individuals with less than a college education (Fig. 2*A*), following refs. 37 and 38. We are able to detect with 90% power a small positive linear trend of a magnitude of 0.05 or greater in absolute value in our event study on firm-level emissions (i.e. a pretrend of such size is likely to generate at least one statistically significant preperiod event study coefficient). Our estimated treatment effect is 0.36 [Table 1 Panel (*A*) column 3], which is seven times as large as the smallest magnitude of pretrend that we can reject with 90% power (0.36/0.05 = 7.2). In *SI Appendix*, Table S14, we repeat this analysis for the event study on emission activities using different SES indicators (the share of the population with less than a high school or college education, the share of rural *hukou* holders, and the share of individuals in skilled occupations) (Fig. 2 and *SI Appendix*, Fig. S3). Our estimated treatment effect is always substantially larger than the smallest magnitude of pretrend in absolute value that we are able to detect with 90% power.

##### Potential heterogeneous treatment effects.

A recent literature shows that, under a continuous treatment setting, the potential heterogeneous treatment effects across units may confound difference-in-differences estimates from a two-way fixed effects (TWFE) model, even when treatment timing is uniform (39–41). We follow ref. 42 to re-estimate the policy’s effects using a binary definition of treatment, which equals one if the share of low-SES individuals in a city is above the median or mean level. The empirical pattern remains consistent: Lower-SES cities experienced significantly less pollution abatement compared to their higher-SES counterparts (*SI Appendix*, Table S15). (Under a setup with a binary treatment variable, where the treatment timing is uniform and treatment status changes at most once, the TWFE estimates are robust to heterogeneous treatment effects (43)).

Two recent papers propose a treatment effect heterogeneity-robust estimator under a two-period setup with continuous treatment—the Weighted Average of Slopes (WAS) estimator (40, 41). To fit the two-period setup in these papers, we limit our sample to the years 2005 and 2010 to form a two-year panel at the city or firm level. *SI Appendix*, Table S16 presents the WAS estimates of the coefficients on the interaction between the low-SES population share and the post-2005 indicator. Columns 1 and 2 use city-level emissions as the dependent variable, and the WAS estimates are close to our baseline TWFE estimates in Table 1. (The results imply that a one percentage point increase in the share of below college (below high school) populations is associated with a 0.28 (0.25) thousand tons reduction in pollution abatement). Columns 3 and 4 show the effects on firm-level emissions: The WAS estimates are statistically significant and larger in magnitude than the corresponding TWFE estimates, likely because we limit our sample to firms that appear in both 2005 and 2010. (Using the two-period firm panel covering 2005 and 2010 to replicate our baseline estimation in Eq. **1**, the TWFE estimate of the coefficient on the interaction between the below-college population share and the post-2005 indicator is 0.84, which is of a similar magnitude to the corresponding WAS estimate reported in *SI Appendix*, Table S16 column 3).

To summarize, we document a robust pattern that the 11th FYP decreased water pollution for all, but more so for higher- relative to lower-SES areas. We note that since higher-SES areas had started out more polluted and experienced greater abatement as a result of the policy, the absolute gap in water pollution exposure between poor and rich areas actually decreased. (*SI Appendix*, Fig. S5 shows the distribution of COD emissions in 2005 and 2010, respectively, by deciles of low-SES population shares in the baseline year of 2000. The figure again demonstrates that cities with a higher share of low-SES individuals experienced smaller emission reductions). However, throughout the entire period of the 11th FYP, low-skilled individuals had much lower access to purified tap water than the high skilled. Moderated by persistent inequitable access to tap water, the differential reductions in pollution emissions based on SES that we find may exacerbate health inequities even with the reduction in absolute pollution gaps. With this in mind, we proceed to examine the policy’s effects on health and labor outcomes in the subsequent section.

### Results on Health and Labor Outcomes.

Various negative health consequences have been associated with water pollution and, as health is an input into labor productivity, these effects may also extend to labor impacts. In this section, we use individual-level longitudinal data to analyze the policy’s impacts on gaps in health and labor outcomes between individuals of high- and low- SES and how the impacts vary with access to defensive infrastructure, i.e. tap water. Individual SES is defined based on skill level (i.e., below college).

We begin by providing descriptive patterns in health and economic outcomes. Table 2 Panel (*A*) reports average gaps in measures of well-being between low- and high-skill individuals before and after the enactment of the 11th FYP, presented separately for individuals with and without access to tap water. Included in each column are *P*-values from *t*-tests on the difference in means of SES-related gaps, conditional on individual FEs. Among those without access to tap water, SES-related disparities in cardiovascular disease (CVD), tumor diagnoses, and the number of inactive days due to illness increased substantially following the policy. Disparities in labor supply (i.e., daily working hours) and wage percentiles also increased in absolute value. Regarding tumor diagnoses, daily work hours, and wage percentiles, the means of these gaps are significantly different between the pre- and postpolicy periods. While the differences in the means of gaps in CVD incidence and inactive days are statistically insignificant, the *P*-values are moderate (around 0.2), suggesting some evidence of change. In contrast, for individuals with access to tap water, gaps in these outcomes decreased slightly in most cases, and the associated differences in means following the policy are all statistically insignificant.

**Table 2.** Impact on health and labor outcomes

	(1)	(2)	(3)	(4)	(5)
Dep. var.:	Cardiovascular disease	Inactive days	Tumor	Daily work hours	Wage percentile
*Panel A: Mean gaps in health and labor outcomes (low-SES – high-SES)*					
Preperiod gaps, without tap water	−0.020	−0.024	0.002	0.067	−4.997
Post-period gaps, without tap water	0.013	0.168	0.005	−0.559	−16.043
*P*-value (Pre vs. post gaps), without tap water	[0.224]	[0.208]	[0.030]	[0.011]	[0.021]
Preperiod gaps, with tap water	0.016	0.063	0.000	0.187	−11.022
Post-period gaps, with tap water	0.004	0.029	−0.003	0.110	−13.305
*P*-value (Pre vs. post gaps), with tap water	[0.853]	[0.671]	[0.321]	[0.993]	[0.298]
*Panel B: Baseline estimates from the DDD model*					
Below college × NoTap × Post_05_($\pi_{4}$)	0.044**	0.270	0.008*	−0.727**	−10.035**
	(0.014)	(0.148)	(0.003)	(0.223)	(2.503)
Below college × Post_05_($\pi_{3}$)	0.002	−0.031	−0.003	−0.034	−0.825
	(0.018)	(0.065)	(0.002)	(0.099)	(2.901)
Estimates of $\pi_{4}+\pi_{3}$:	0.046	0.239	0.004	−0.761	−10.860
*P*-value of $\pi_{4}+\pi_{3}$:	[0.169]	[0.188]	[0.011]	[0.012]	[0.053]
Observations	25,097	25,097	28,936	4,801	3,337
Mean of dep. var.	0.111	0.160	0.004	7.823	58.024
SD of dep. var.	0.314	1.238	0.066	1.850	27.784
Individual FE	X	X	X	X	X
Province × Year FE	X	X	X	X	X

*Notes:* Panel (*A*) reports *P*-values from *t*-tests on the difference between the preperiod and post-period means of SES-related gaps, conditional on individual fixed effects. Panel (*B*) presents estimates from individual level regressions. We drop observations with missing values in dependent variables, tap water access and education. Two-way robust SEs clustered at province and year levels are reported in parentheses.

***P< 0.01, **P< 0.05, *P< 0.1.

#### Baseline results on health and labor outcomes.

Table 2 Panel (*B*) reports our estimates of the DDD model (Eq. **3**). All columns include individual and province-by-year fixed effects. The interaction of the below-college indicator and the post-2005 dummy ($\pi_{3}$) is always insignificant, whereas the coefficient estimates of the triple interaction term between the below-college indicator, the post-2005 dummy, and the no tap water access dummy are often statistically significant ($\pi_{4}$). This suggests that the effects of the policy on disparities in health and labor outcomes are different between those with and without access to tap water. The sum of the coefficients on the double interaction ($\pi_{3}$) and the triple interaction ($\pi_{4}$) captures the policy’s impact for individuals without access to tap water. Panel (*B*) also reports the *P*-value associated with the joint estimate of $\pi_{3}+\pi_{4}$.

Consistent with the descriptive pattern, our DDD estimates document increased gaps in health and labor market outcomes among individuals who do not have access to tap water. Differences in the diagnoses of cardiovascular disease and tumor by skill level increase by 4.6 percentage points (pp) (*P*= 0.17) and 0.4 pp (*P*= 0.01), respectively. The policy impacts correspond to a 0.15 and 0.06 SD increase from baseline disparities in diagnosis rate for CVD and tumor, respectively. The gap in the number of inactive days due to illness increased by 0.24 d (*P*= 0.19) and daily hours worked last week increased by 0.76 (*P*= 0.01) in absolute value. (This is consistent with the descriptive pattern reported in Table 2 Panel (*A*). Among those without access to tap water, low-SES individuals experienced slightly fewer inactive days and worked marginally longer hours per day prior to the policy compared to their high-SES counterparts; following the implementation of the policy, they had more inactive days and fewer daily working hours relative to high-SES individuals). We define wage rankings based on the percentile of real wages and find that the gap in wage ranking increased by 10.86 percentiles in absolute value for those without access to tap water. [As shown in Table 2 Panel (*A*), among those without access to tap water, low-SES individuals always earned less than their high-SES counterparts, with the earnings gap widening after the policy was implemented]. [Firms may offer higher wages to compensate for the risks of pollution in order to keep people working in polluted places. However, given high mobility costs (prior to 2010) associated with the *hukou* system and limited information about local pollution, hedonic wage differentials may not reflect pollution differentials in China (44, 45)]. Importantly, we do not find evidence that gaps in well-being are impacted among those with access to tap water. If the marginal damages from pollution were constant across SES groups and between those with and without access to tap water, the policy-driven changes in health disparities would not be systematically related to the availability of tap water. Nevertheless, we observe that the policy widened health disparities between SES groups among individuals without access to tap water while there was no meaningful effect for those with tap water access. These results suggest that heterogeneous damages from pollution exposure play an important role in the increase in health gaps and that the change in gaps is unlikely to be purely driven by differences in the levels of pollution reduction.

Our results demonstrate that health and labor4iffer significantly by access to tap water, highlighting the policy impacts through changes in water pollution. In *SI Appendix*, Fig. S6, we plot an event study of the policy impacts over time. While we only have two prepolicy waves from the CHNS data (2000 and 2004), we do not find evidence of preexisting trends in health or wage rankings.

Taken together, the spatially differentiated abatement in water pollution did not meaningfully affect gaps in health and labor market outcome by skill for those with tap water access, but significantly widened these gaps for those without access. The 2005 Population Census of China shows that up to 543 million unskilled individuals did not have access to purified water. The barrier for the poor to access purified water was large and existed throughout the entire period of the 11th FYP. Despite overall reductions in water pollution, uneven implementation of abatement, combined with persistent, unequal access to clean drinking water, exacerbated the inequality in health and labor market outcomes in China.

#### Sensitivity analysis of health and labor outcomes.

##### Other water pollution policies.

We first examine whether our estimated health and labor market consequences are driven by other concurrent water pollution regulations over the same period. Specifically, we add various controls to address the potential confounding effects of three important water pollution control policies–*The Water Pollution Prevention and Control Plan for Major River Basins (2006–2010)*, *The River Chief Policy*, the release of *The List of National Key Monitoring Firms*. [In *SI Appendix*, Table S17 Panel (*A*), we create an indicator for cities in protected areas covered by *The Water Pollution Prevention and Control Plan* and control for interactions between the indicator and year fixed effects. In Panel (*B*), we control for an indicator for whether a city had implemented *The River Chief Policy* in a particular year. In Panel (*C*), we control for a city’s total number of *Key Monitoring Firms*]. The results are presented in *SI Appendix*, Table S17. Our results are similar to before.

##### Additional controls and placebo tests.

Health outcomes vary across socioeconomic groups, and these disparities may widen with age. Since we use individual-level longitudinal data, the post-2005 indicator may capture age-related differences. Additionally, access to tap water may be correlated with broader health and environmental infrastructure, which may influence how health conditions evolve over time. In particular, when individuals have access to tap water, health disparities across socioeconomic groups may follow a different age pattern, potentially due to correlated factors like access to hospitals and medical services. To address this concern, *SI Appendix*, Table S18 Panel (*A*) repeats our triple differences analysis with additional controls for interactions between measures of health and medical services (hospital beds per capita, doctors per capita), the low-SES indicator, and the post-2005 indicator. Adding these controls does not change our empirical pattern. Thus, our results are unlikely to be driven by health disparities arising from factors correlated with tap water access.

*SI Appendix*, Table S18 Panel (*B*) further examines potential confounding effects associated with age. We control for interactions between age fixed effects and the low-SES indicator, as well as interactions between age fixed effects and the indicator for no tap water access. The results show a similar pattern, suggesting that age-related variation in health outcomes is unlikely to confound our findings.

We next perform placebo tests based on access to an indoor bathroom. Such access likely reflects household economic conditions and may also be associated with broader health infrastructure. Moreover, access to an indoor bathroom can affect health outcomes through channels other than water pollution–for example, by reducing the spread of infectious diseases. *SI Appendix*, Table S19 Panel (*A*) alleviates the concern about nonwater pollution channels and finds that the 11th FYP did not meaningfully affect disparities in health outcomes between those with and without access to indoor bathrooms. We also perform additional placebo tests by randomizing individual access to tap water or skill level (*SI Appendix*, Table S19 Panels *B* and *C*). Our estimated effects on the gaps for those without access to tap water disappear.

##### The effects of changes in air pollution.

The 11th FYP also targeted air pollution in addition to water, and set $SO_{2}$ reduction mandates for different regions (31). In *SI Appendix*, Table S20, we re-estimate the triple differences model on health outcomes that are unlikely to be related to water pollution. Notably, we do not find evidence that the gap in asthma and the incidence of respiratory disease increase in response to the policy, which makes air pollution an unlikely pathway of effect. (We also find no evidence that the policy changed the differential likelihood of muscle aches pains or ear, nose, and throat (ENT) diseases between individuals with high vs. low skill. Moreover, the impacts on this set of outcomes do not differ based on tap water access). Re-estimating the baseline DDD model to control for city-level SO_2_ and PM2.5 also does not meaningfully change our estimates and is again supportive of water pollution being the predominant pathway of effect (*SI Appendix*, Table S21). The results are consistent with our earlier finding that the 11th FYP did not differentially impact firm-level SO2 emissions between areas of low and high disadvantage.

##### Multiple hypothesis testing.

In Table 2, we examine the policy’s impact on a range of health and labor market outcomes. To address concerns about overrejection of null hypotheses, we conduct multiple hypothesis testing. *SI Appendix*, Table S22 presents Romano and Wolf *P*-values corresponding to the coefficients in Table 2. The Romano and Wolf *P*-values for our primary variable of interest—the interaction between the below-college indicator, the post-2005 indicator, and the no tap water indicator—fall below 10% across all outcomes except for inactive days.

##### Differential changes in tap water access.

One might expect that among people without tap water in 2005, high-SES individuals experienced greater improvements in tap water access than their low-SES counterparts. These potentially differential changes in tap water access by SES could confound our estimated effect on health disparities. However, this is not the case in our context. *SI Appendix*, Fig. S7 presents the proportion of individuals without tap water access in each city in 2010 against that in 2005, separately for those with college education (the *Left* panel) and those without (the *Right* panel). For both low-SES and high-SES groups, the proportion of individuals without tap water access at the city level remains generally consistent over the 2005–2010 period.

To further examine this concern, we regress changes in the share of individuals with the coverage of tap water in each city between 2005–2010 on low-SES population shares at baseline and city-specific COD emissions abatement targets under the 11th FYP. *SI Appendix*, Table S23 shows that baseline SES composition of the population and city-level emission reduction mandates are not statistically different from zero, and the R-squared is below 0.02 across all specifications. Therefore, baseline low-SES population shares and COD reduction targets cannot predict changes in access to tap water during the 11th FYP.

Additionally, we use five waves of CHNS data from 2000 to 2011 to calculate the community-level tap water coverage rate separately for low- and high-SES individuals. (Some communities include both low- and high-SES individuals. In such cases, we calculate the share of low-SES individuals with access to tap water in a given community, as well as the share of high-SES individuals with access to tap water in that community). *SI Appendix*, Table S24 shows that changes in tap water coverage during 11th FYP were statistically indistinguishable between the two SES groups.

Together, these checks limit the concern that differential changes in tap water access between low-SES and high-SES individuals during the period of 11th FYP drive our triple-difference results. *SI Appendix*, Table S25 further shows that, within a city, baseline access to tap water does not predict changes in 5-y average COD emissions following the policy, nor changes in annual COD emissions between 2006 and 2010, 2001 and 2010, or 2001 and 2005. Our analysis documents that the policy’s effects on health disparities are systematically different for individuals with and without access to tap water. However, we caveat that while we demonstrate that potential confounders such as access to health and medical services are unlikely to confound our results, it is possible that remaining unobservables correlated with tap water access could result in differential marginal damages of water pollution. Our placebo tests show that access to other infrastructure—likely associated with these unobservables—does not drive the differential health effects of the policy. Nevertheless, we interpret our findings cautiously in light of this caveat and acknowledge that our results should not be interpreted as definitive evidence of a causal role for tap water access, but rather as evidence suggesting that the policy’s implications depend on individuals’ vulnerability to water pollution.

##### The migration response to pollution changes.

Finally, migration as a pollution avoidance strategy may be an alternative channel through which the policy affects health and labor outcomes (46). We evaluate whether sorting in response to the policy drives the well-being gaps that we measure. We find no evidence that the policy differentially changed the tendency to move for the unskilled relative to the skilled group (*SI Appendix*, Table S26).

## Discussion

China’s 11th FYP included significant regulation to mitigate water pollution across the country. Because pollution reduction mandates differed by regions, regulations were likely to have distributional consequences on pollution exposure and well-being beyond overall effects. Indeed, while coastal cities where wealthier households resided were exposed to higher levels of COD emissions before the 11th FYP, they experienced a greater reduction in emissions during the policy period.

In this paper, we estimate the differential impact of this nationwide policy on firm COD emissions in higher vs. lower socioeconomic (SES) areas. We find that the policy reduced COD emissions everywhere, but less so for cities with higher shares of low SES individuals. We then extend the analysis to examine the policy’s effect on health and labor market outcomes of individuals with high relative to low socioeconomic disadvantage. We generally find that the policy had an unintended distributional consequence of widening gaps in health, wage, and labor supply between low relative to high skill individuals, particularly for the population without access to tap water.

China has made significant progress in limiting industrial water pollution in recent years (23). These gains have been enabled by the implementation of large-scale policies, founded upon the lessons learned from decades of policy experimentation at more local, regional levels (47). The overall impact of a policy, however, can be underscored by significant heterogeneity. Our paper documents how policy-driven changes in pollution can interact with unequal access to defensive infrastructure to increase health and economic inequities. This moderating role of defensive infrastructure and socioeconomic status in determining policy impacts across regions also highlights a limitation of scaling up policies based on policy experimentation within regions (47).

Our study can be viewed as an example where a policy improved water pollution for all (as predicted), but interregional differences in socioeconomic status and defensive infrastructure resulted in unintended impacts on the distribution of benefits. In this way, we provide empirical support for both government action and firm compliance as causal mechanisms that contribute to health and economic inequity. Our assessment of subsequent health and labor supply impacts also complement previous work to understand the equity consequences of environmental policies on health and economic well-being. Our analysis proposes an environmental health channel, with interactions with the built environment, to explain the enduring within-country gaps in welfare across the developing world.

## Materials and Methods

### Data.

#### Firm-level emissions and establishment data.

Firm-level emissions data come from the Environmental Survey and Reporting (ESR) database, which is a large administrative dataset collected by the MEP of China. The ESR covers all top firms/plants contributing 85% of total emissions of the major pollutants in a county. These major pollutants are specified by the MEP and include COD, SO_2_, ammonia nitrogen, industrial smoke and dust, and solid waste. Thus, ESR contains all local major industrial emissions sources in China, like heavily polluting industrial firms, urban sewage treatment plants, and hospitals. These key emitters are required to report detailed information on major pollutant emissions along with other firm attributes.

Despite being self-reported by firms, the ESR data are subject to multiple layers of scrutiny. Local Environmental Protection Bureaus (EPBs) conduct unannounced inspections and monitoring activities to ensure the accuracy of the data. Once validated, the data are compiled into reports and submitted to higher-level authorities, including provincial EPBs and the MEP. These upper-level agencies further verify the data through random spot checks, onsite inspections, flight inspections, and cross-checks. This multitiered oversight system enhances data reliability and prevents potential manipulation by firms. For firms with complex administrative structures, such as joint enterprises, their branch plants are treated as the basic units for reporting pollution information. Additionally, China’s Environmental Protection Law prohibits using ESR data to penalize or regulate polluting firms. Consequently, firms included in the ESR sample have little incentive to misreport their emission records. This legal safeguard aims to ensure data accuracy, making ESR a more reliable source for policy-making rather than enforcement (19).

In this paper, we employ the longitudinal firm-level emissions data of the ESR dataset from 2001 to 2010. We clean the sample to remove firms with missing addresses and no COD emissions throughout the entire sample period. Our baseline panel data track about 127,000 water pollution firms during this period, and use annual COD discharge to measure firm-level water pollution.

#### Population census data.

We measure baseline city-level demographic features using the 2000 Population Census of China. The census data contain a wide range of variables about demographic and economic characteristics of individuals, such as age, gender, educational attainment, hukou type (rural/urban), access to tap water, and residential address. We use these data to categorize a city’s baseline social and economic disadvantage in the main analysis on firm behavior. We supplement our analysis with the 2005 and 2010 Censuses for tests in our robustness checks. *SI Appendix*, Fig. S8 illustrates spatial distribution of baseline demographic characteristics in 2000.

#### Health, wage, and labor supply data.

We obtain individual-level data on health, labor supply, and wages from the China Health and Nutrition Survey (CHNS) (48). We use five waves of survey from 2000 to 2011 (i.e., 2000, 2004, 2006, 2009, and 2011), linking households and individuals across time to allow for longitudinal analysis. The survey covers households and individuals across 49 cities that vary in economic prosperity, public resources, geographic characteristics, and health indicators. The survey records a vast array of demographic and health characteristics of individuals, including age, education levels, employment details, labor supply, wage, health status, and illness. *SI Appendix*, Fig. S9 illustrate the geographic distribution of the 9 provinces covered by the CHNS data between 2001 to 2011. (The CHNS records the average monthly wage received by respondents in the year proceeding the survey year, but collects information on health outcomes and labor supply in the survey year).

We focus on the sample of individuals with recorded values of tap water access and education, and restrict our data to those who appear both before and after China’s 11th FYP in the survey. Within this sample, health and illness are surveyed for most individuals, but wage and labor supply information are collected for a smaller subset. We drop individuals who are missing information on health and labor outcome, and provide evidence in *SI Appendix* showing that the probability of having missing values (across different dependent variables) is not systematically associated with our main effects of interest. Specifically, we show in *SI Appendix*, Table S27 that the main coefficient in our triple differences specification is not statistically significant. *SI Appendix*, Table S28 reports summary statistics of the key variables used in the analysis. We provide details on all datasets in *SI Appendix*

### Empirical Methods.

#### Firms’ polluting activities.

We first examine whether water pollution regulation in China’s 11th Five-Year Plan disproportionately changes firm-level COD emissions for areas of high and low disadvantage. Using firm-level emission data from 2001 to 2010, we estimate the following difference-in-differences (DD) specification:

[1] $$\begin{array}{c} Emission_{\mathrm{ict}}=\phi Demographic_{c,2000}\times Post_{t}+\nu_{i}+\gamma_{p,t}+\epsilon_{\mathrm{it}} \end{array}$$

$Emission_{\mathrm{ict}}$ is the amount of COD emissions (in tons) of firm i in city c during year t. $Demographic_{c}$ represents baseline city-level population shares (in percent) of a specific demographic characteristic in 2000 and serves as a continuous measure of treatment. There is not a clearly defined group of cities or individuals that is “untreated” by the policy. Instead, our analysis examines the heterogeneous impacts of the policy based on an area or an individual’s SES. The demographic characteristics are evaluated at the beginning of the decade to avoid the issue of contemporaneous demographics responding to the policy. (*SI Appendix*, Fig. S10 shows that the share of disadvantaged groups at the city level is generally persistent over the 2000–2010 period). Our baseline analysis defines SES based on educational attainment. Specifically, we leverage the concentration of individuals without a college degree (including college dropouts) and those without a high school degree (including high school dropouts). $Post_{t}$ is a dummy variable that indicates the post-treatment period of the policy (2006–2010), i.e., $Post_{t}=1\;\forall t>2005$ and 0 otherwise. Our specification follows refs. 17 and 18, who evaluate the effects of water pollution regulation under the 11th FYP by comparing changes in regional pollution activity before and after 2006.

The parameter of interest, ϕ, measures the *relative* change in firm-level COD emissions driven by the 11th FYP in cities with a higher concentration of a given demographic group vs. cities with a lower concentration of that group. In other words, we examine whether China’s nationwide pollution regulation induces differential changes in water pollution between more and less disadvantaged areas. We include firm fixed effects ($\nu_{i}$) to remove time-invariant differences across firms in lower- and higher- SES areas that may contribute to the gap in water pollution. We add province-by-year fixed effects ($\gamma_{p,t}$) to account for province-level trends in firm activity and differences in public policies across regions or provinces that may affect firm emissions. [We evaluate the parallel trends assumption needed for causal inference with a series of event study regressions to examine differential pollution emission trends based on the baseline population’s SES composition in the years leading up to the policy (Fig. 2 and *SI Appendix*, Fig. S3)].

Next, we study how China’s spatially differentiated pollution control policy affects the agglomeration of polluting firms across cities with different baseline fractions of disadvantaged groups. We use the entry and exit of polluting firms recorded in the ESR database as outcome variables and estimate the following city-level regression:

[2] $$\begin{array}{c} Num_{\mathrm{ct}}=\phi Demographic_{c,2000}\times Post_{t}+\theta_{c}+\gamma_{p,t}+\epsilon_{\mathrm{ct}}, \end{array}$$

where $Num_{\mathrm{ct}}$ represents the number of polluting firm entrants or exits in city c in year t, and city fixed effects $\theta_{c}$ capture time-invariant, city-specific characteristics. The coefficient of interest ϕ compares the changes in firm entry or exit driven by the 11th FYP in cities with a higher concentration of a given demographic group vs. cities with a lower concentration of that group. For our baseline analysis of emission activities and individual health and labor outcomes, we use two-way cluster-robust SEs at the province and year levels to account for potential correlation of the error term within provinces or within years. Our results are robust to different error term structures that we assume for inference. In *SI Appendix*, Tables S29 and S30, we perform wild cluster bootstrapping procedures given the small number of clusters, and this yields very similar results.

#### Inequities in health and labor outcomes.

Pollution regulation in the 11th FYP reduced overall levels of COD emissions across the country, but regions with higher concentrations of disadvantaged groups experienced significantly less pollution abatement. Moreover, disproportionate access to purified drinking water may interact with changes in pollution to exacerbate inequity in health outcomes. Studies in toxicology and epidemiology have identified both cancer and noncancer risks associated with drinking water contamination, including anemia, reproductive system problems, nervous system problems, and cardiovascular disease (49). Such health insults may further detract from an individual’s ability to work or job performance.

Our aim is to estimate the 11th FYP’s impact on gaps in health and labor market outcomes between low-SES and high-SES individuals through the mechanism of water pollution. This is complicated by other concurrent policies, including air pollution regulations in the 11th FYP. Specifically, the 11th FYP also placed pollution reduction targets on SO_2_, which may contribute to gaps in well-being. Moreover, the policy’s effects depend not only on the extent of pollution abatement experienced by each SES group but also on the vulnerability of individuals to pollution exposure. Due to substantial disparities in tap water access, high- and low-SES individuals likely face different marginal damages from water pollution, resulting in uneven benefits from pollution abatement.

We next estimate a triple difference model by comparing differential changes in health gaps across SES groups between those with and without tap water access. This framework allows us to identify the portion of health disparities attributable to changes in exposure to water pollution and unequal protection provided by defensive infrastructure. The specification is given by

[3] $$\begin{array}{cc} Y_{j,t}= & \pi_{0}NoTap_{j,t}+\pi_{1}Demographic_{j}\times NoTap_{j,t}\\ & +\pi_{2}Post_{t}\times NoTap_{j,t}+\pi_{3}Demographic_{j}\times Post_{t}\\ & \pi_{4}Demographic_{j}\times Post_{t}\times NoTap_{j,t}+\delta_{j}+\gamma_{p,t}+\epsilon_{\mathrm{jt}}, \end{array}$$

where $Y_{j,t}$ represents individual j’s health status and labor outcomes in year t, and $\delta_{j}$ denotes individual fixed effects. The health outcomes we use include indicator variables for whether the individual is suffering from a tumor (In the CHNS data, the definition of “tumor” includes both malignant and benign tumors) and cardiovascular disease. These indicators take the value of one if individual j suffers from the particular illness in year t, and zero otherwise. We also measure overall health by the number of days that an individual cannot perform his or her routine daily activities (in the month prior to the survey) due to illnesses. For labor outcomes, we use daily working hours and wage ranking based on percentiles of the real wage distribution. [Real wages are calculated in 1999 constant prices by deflating nominal wages using the Consumer Price Index (CPI)]. We characterize individuals based on education because skill level is less likely to change over time in the short to medium term (compared to occupations and hukou types). The policy-induced changes in gaps in health status and labor outcomes between high- and low-skill individuals likely depend on their access to tap water. The coefficients $\pi_{3}+\pi_{4}$ represent the resulting changes in health and economic gaps for individuals without access to tap water (i.e., $NoTap_{j,t}=1$), while $\pi_{3}$ captures changes in these gaps for those with tap water access. The parameter of interest is the triple interaction term $\pi_{4}$, which captures how the impact of the policy on the well-being gap differs between individuals without and with access to tap water. (A causal effect relies on an assumption that the gap in outcomes by skill level for those without tap water access would be parallel to the gaps by skill level for those with tap water access had the policy not been implemented. *SI Appendix*, Fig. S6 examines this assumption, showing that prior to the 11th FYP, gaps in health and wages by skill did not trend differently between those with and without access to tap water).

## Supplementary Material

Appendix 01 (PDF)

## Data Availability

Code needed to reproduce the analyses has been deposited at: https://doi.org/doi:10.7910/DVN/CHI8WN (50). We have made all analysis code publicly available (https://doi.org/10.7910/DVN/CHI8WN) (51). However, we are not authorized to publicly share all of the data because some datasets used in this paper are restricted-access. Researchers may request permission to access the same data through the appropriate channels. We have provided details on how to apply for access to these datasets in *SI Appendix*.

## Footnotes

^*^COD is a broad indicator of organic water pollution, closely associated with elevated Biochemical Oxygen Demand (BOD) and toxic substances. High COD levels reduce dissolved oxygen in water, potentially leading to favorable conditions for anaerobic bacteria and harmful microbial communities. COD serves as a proxy for untreated industrial and domestic wastewater, including industrial discharges and sewage, that may carry heavy metals, pathogens, and toxic chemicals.

^†^An average COD-polluting firm discharged 35.7 tons of COD in 2005. Under China’s 11th FYP, COD-polluting firms were required to comply with emission standards and adhere to designated discharge targets. The government also introduced a series of regulations that strengthened pollution control and technological upgrading, promoted wastewater recycling, and phased-out some small-scale, high-pollution enterprises.

^‡^Access to treated tap water can significantly reduce the negative health effects of water pollution by limiting exposure to pathogens and toxic substances. Data from CHNS show that 43% of the population still lacked access to tap water in China in 2009.

^§^Note that while these raw trends were influenced by the water pollution control policy, they may also be affected by other factors impacting health and labor market outcomes.

^¶^By combining the coefficient on the post-2005 dummy (−16.43) and its interaction with the below-college population share (0.17), the results also show that an average city (with the mean below-college population share of 91.02%) experienced a reduction in COD emissions of 1.4 thousand tons (16.426 to 0.165 × 91.021).

^#^The coefficient estimate of the post-2005 indicator implies that, in a hypothetical city where the share of below-college individuals is zero, city-level COD emissions would decline by 16.43 thousand tons following the policy.

^‖^Based on Table 1 Panel (*C*), mean firm emissions decreased from 34.8 tons in the preperiod to 32.13 tons in the post-period.

^**^About 7.6% of firms reported zero emissions in a particular year between 2001 and 2010. The likelihood of a firm reporting positive emissions increased from 85% to 98% after the implementation of the policy. Before the 11th FYP, some polluting firms may temporarily suspended emissions (having zero recorded emissions) due to short-term regulatory pressures but resumed their emissions later. The pollution control policy in the 11th FYP may lead such firms to exit the market permanently. *SI Appendix*, Table S5 analyzes the consequences of the policy at the intensive and extensive margins. Conditional on having positive COD emissions, firms in cities with a larger concentration of low-SES individuals also saw a significantly smaller reduction in emissions (columns 2 to 3). Column 4 examines changes in emissions per unit of output (i.e. the gross industrial output value at the firm-year level).

^††^The specification of our event study on firm emissions is $Emission_{\mathrm{ict}}=\sum_{t=-4}^{-1}\theta_{t}Demographic_{c,2000}\times Pre_{t+2005}+\sum_{t=1}^{5}\phi_{t}Demographic_{c,2000}\times Post_{t+2005}+\nu_{i}+\gamma_{p,t}+\epsilon_{\mathrm{it}}$, where $Pre_{t+2005}$ represents an indicator for year t+2005 (t=−1,..,−4), $Post_{t+2005}$ represents an indicator for year t+2005 (t=1,..,5), $\gamma_{p,t}$ represents province-by-year fixed effects, and $\nu_{i}$ represents firm fixed effects.

^‡‡^*SI Appendix*, Fig. S3 presents an event study of the treatment effect based on alternative indicators of baseline SES (i.e., the share of rural hukou holders or the share of individuals in skilled occupations). We find similar results.

^§§^The MEP ranked polluting firms based on their emissions of several major pollutants. Firms are included in this list if their total emissions of a particular pollutant account for 65% of a given industry.

^¶¶^According to Panel (*C*) of Table 1, an average city experienced an increase in firm exit after the 11th FYP was enacted.

^##^We categorize cities based on an inverse-covariance weighted summary index of various SES measures: the share of individuals without a college degree, the share of individuals without a high school degree, the share of rural hukou holders, and the share of individuals in unskilled occupations (33).
