# Supplementary material for: A spatially differentiated water pollution policy leads to economic and health inequity
Source: Proc Natl Acad Sci U S A. 2025 Sep 24;122(39):e2421404122. doi: 10.1073/pnas.2421404122 (PMC12501171; doi:10.1073/pnas.2421404122)
Supplement: Supplementary file 1 — Appendix 01 (PDF) [file pnas.2421404122.sapp.pdf]

Supporting Information for

A Spatially-differentiated Water Pollution Policy Leads to Economic and Health Inequity

Zhonghui Luo, Lala Ma, Rui Xie and Ran Song

Corresponding Author: Ran Song.
E-mail: ran.song@nus.edu.sg.

This PDF file includes:

Supporting text
Figs. S1 to S10
Tables S1 to S30
SI References

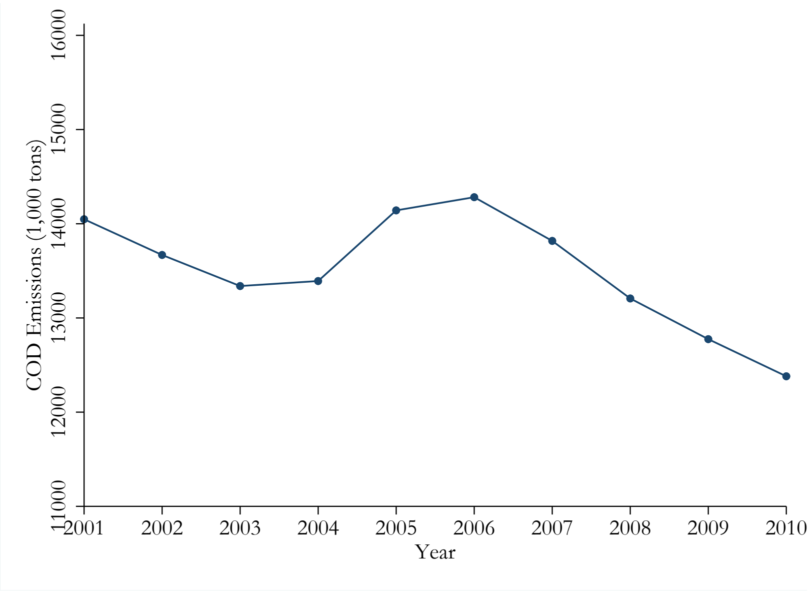

Fig. S1. Total COD Emissions (2001-2010). Notes: This figure shows the trend in total amount of COD emissions in China from 2001 to 2010. Data come from China Statistical Yearbook on Environment.

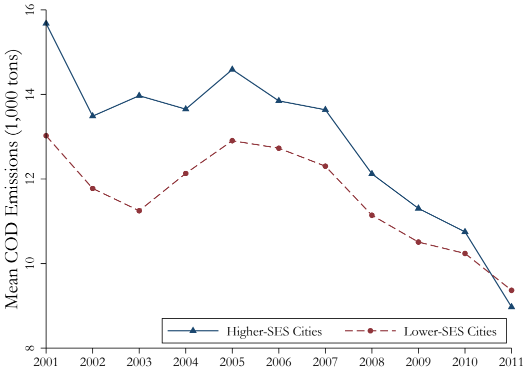

(A) Trends in COD Emissions Exposure in Higher- and Lower- SES Cities

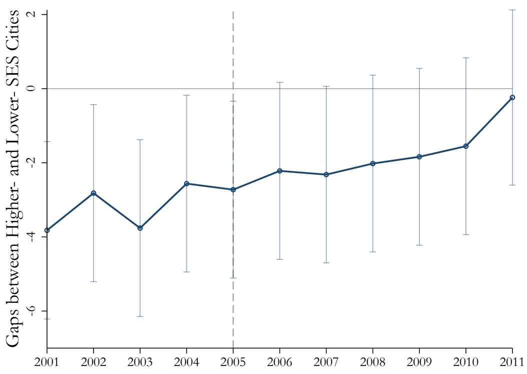

(B) Gaps in COD Emissions between Higher- and Lower- SES Cities

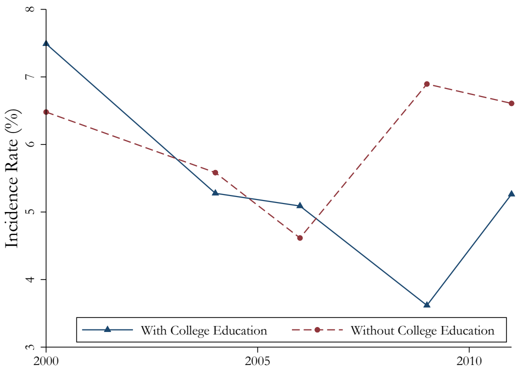

(C) Trends in Incidence Rate of Cardiovascular Disease

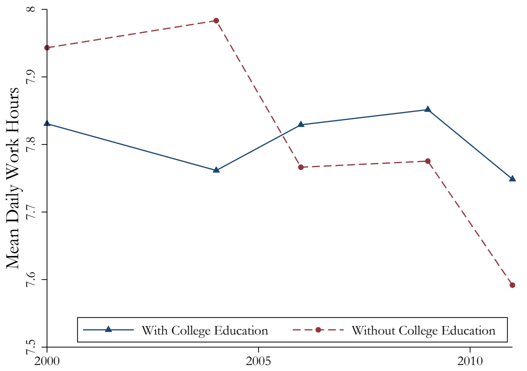

(D) Trends in Daily Work Hours

Fig. S2. Trends in COD Emissions and Health Outcomes. Notes: Fig. S2B shows the differences in mean COD emissions between two city groups (higher- and lower-SES cities) in each year, conditional on province fixed effects. In particular, we regress city-level COD emissions on an indicator for lower-SES cities, as well as interactions between this indicator and dummy variables for the years from 2002-2011, controlling for province fixed effects. The differences in mean COD emissions between the two city groups are calculated based on the coefficient estimates of the lower-SES indicator and these interaction terms. Vertical bands represent 90% confidence intervals in Fig. S2B. To demonstrate trends in health status, Fig. S2C presents new cases of cardiovascular disease. Data come from the Environmental Survey and Reporting (ESR) and the China Health and Nutrition Survey (CHNS).

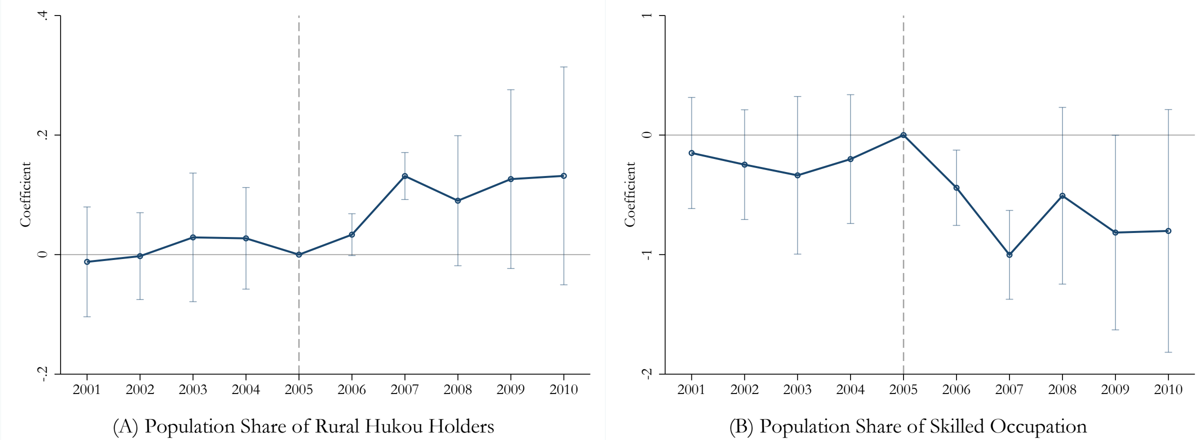

Fig. S3. Firm Behavior Event Study (DD), Higher vs. Lower SES Areas (Alternative Measures of SES). Notes: We test for pre-existing trends and dynamics of the policy in an event study framework. The baseline year of comparison is 2005 (the year before the 11th FYP), and the x-axis shows the year relative to that baseline year. Vertical bands represent 95% confidence intervals. Data come from the Environmental Survey and Reporting (ESR) and China Population Census.

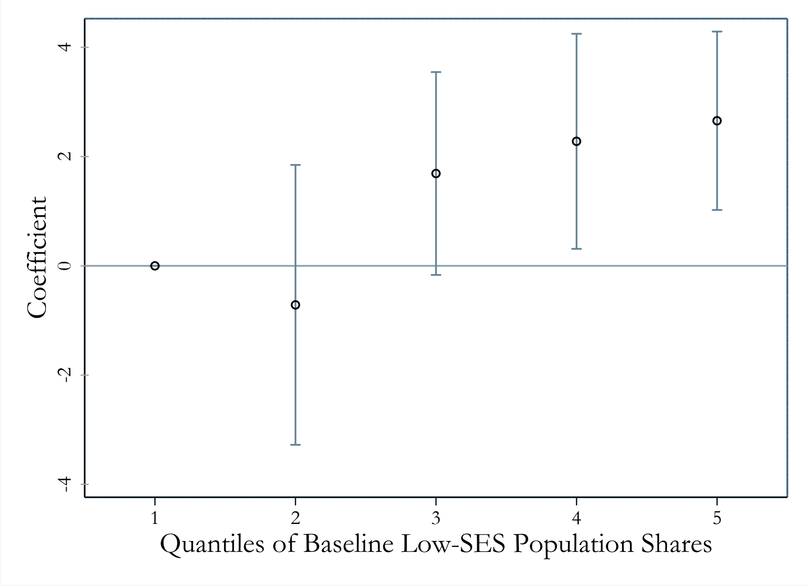

Fig. S4. Effects by the Quartiles of Baseline SES. Notes: We categorize cities into five groups based on the quantiles of a city's baseline SES and regress city-level COD emissions on four interaction terms—specifically, interactions between the post dummy and the second, third, fourth, and fifth quantile dummies, respectively. In particular, we construct an inverse-covariance weighted summary index of various SES measures: the share of individuals without a college degree, the share of individuals without a high school degree, the share of rural *hukou* holders, and the share of individuals in unskilled occupation. The specification is $Emission_{ct} = \sum_{n=2}^{5} \phi_n Quantile_{c,n} \times Post_t + \theta_c + \gamma_{p,t} + \epsilon_{ct}$, where $Quantile_{c,n}$ is an indicator for the *n* th quantile of a city's baseline SES, θ_c is city fixed effects, and $\gamma_{p,t}$ is province-by-year-fixed effects.

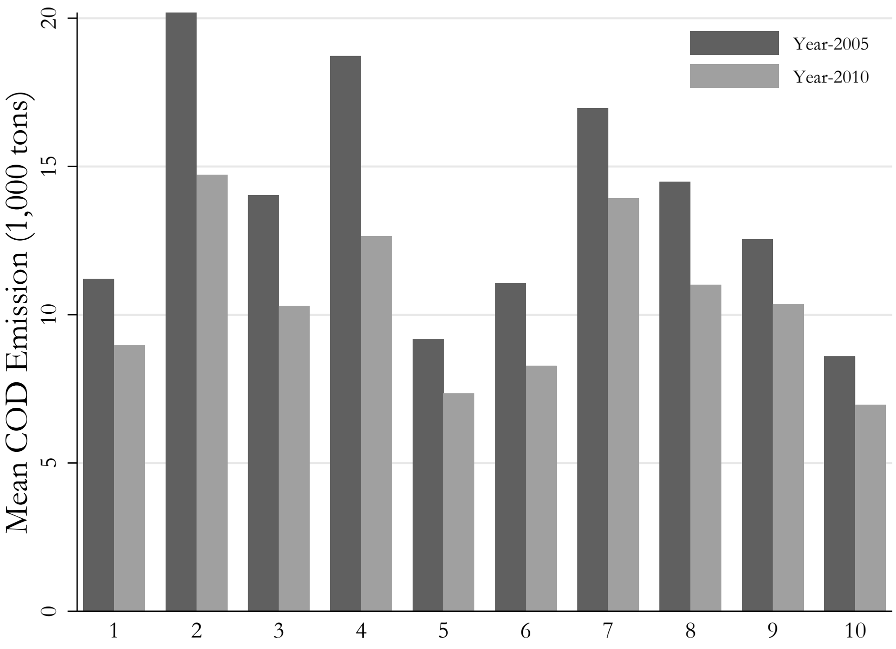

Fig. S5. Distribution of COD Emissions by Decile of low-SES Population Shares (2005-2010). Notes: We aggregate firm-level COD emissions to the city-by-year level. We create deciles of unskilled population shares in the baseline year of 2000 and calculate average COD emissions at each decile in 2005 and 2010, respectively. Unskilled people are those who do not have a college degree. Data come from the Environmental Survey and Reporting (ESR) and China Population Census.

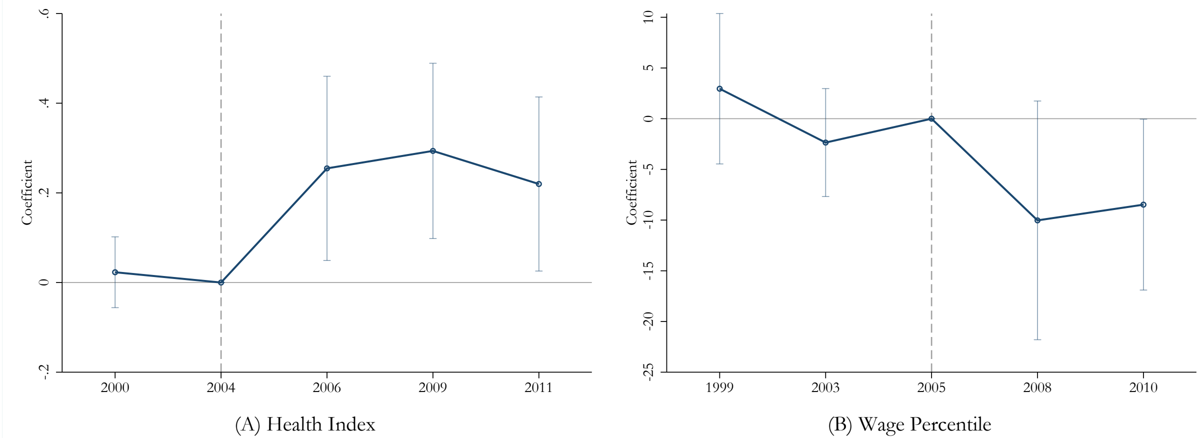

Fig. S6. Health and Labor Impacts Event Study (DDD), High vs. Low SES Individuals. Notes: We test for pre-existing trends in health and labor outcomes using an event study framework. The outcomes we examine are an overall health problem index (A) and wage percentile (B) . The CHNS records the average monthly wage received by respondents in the year proceeding the survey year. The baseline year of comparison is 2004 for the overall health problem index and 2005 for the wage percentile, and the x-axis represents the years relative to that baseline year. In the panel (A), we construct an inverse-covariance weighted summary index of different health outcomes: the incidences of tumor and cardiovascular disease, and the number of days that an individual cannot perform his or her routine daily activities due to illnesses. Vertical bands represent 95% confidence intervals. Data come from the China Health and Nutrition Survey (CHNS).

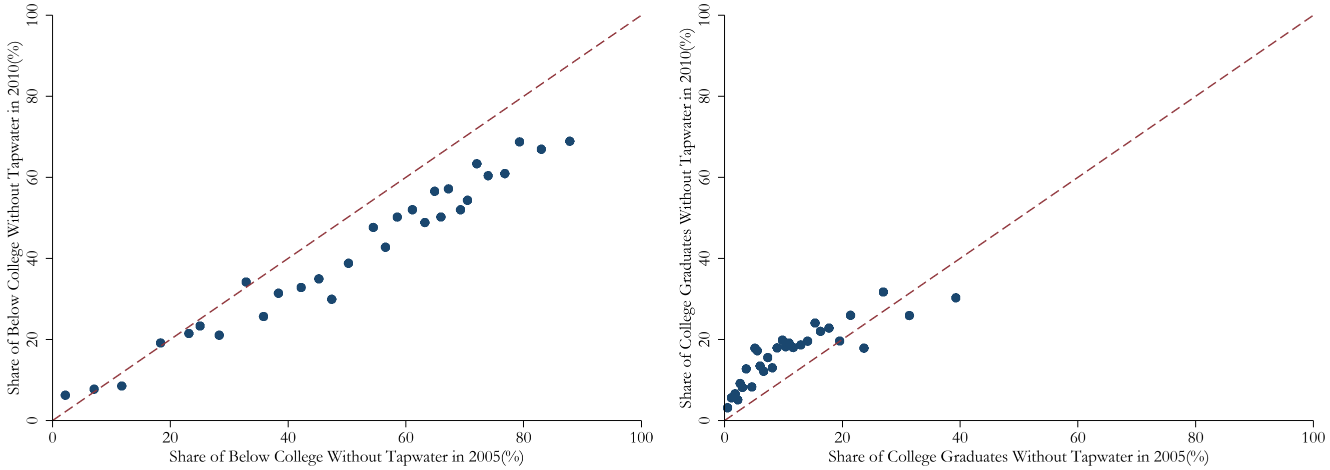

Fig. S7. Persistence of tap water access during 2005-2010. Notes: This figure plots the share of individuals without tap water access in 2010 against their values in 2005 for those without a college degree (left panel) and those with a college degree (right panel). We divide cites into 30 groups based on the quantile of the share of individuals without tap water access in 2005, separately for those with and without a college degree. The x-axis denotes the mean value of the share of individuals without tap water access in each quantile in 2005, and the y-axis denotes the mean value of the share of individuals without tap water access in each quantile in 2010. Data come from the China Population Censuses.

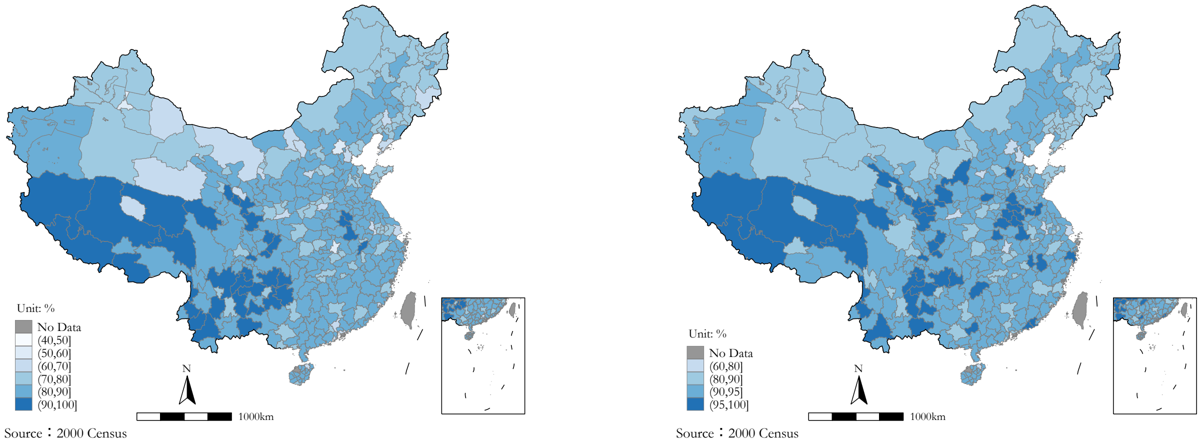

(A) Share of Below HS

(B) Share of Below College

Fig. S8. Spatial Distribution of Baseline Demographic Characteristics in 2000. Notes: (A) Spatial distribution of population share without high school education. (B) Spatial distribution of population share without college education. Data come from China Population Census.

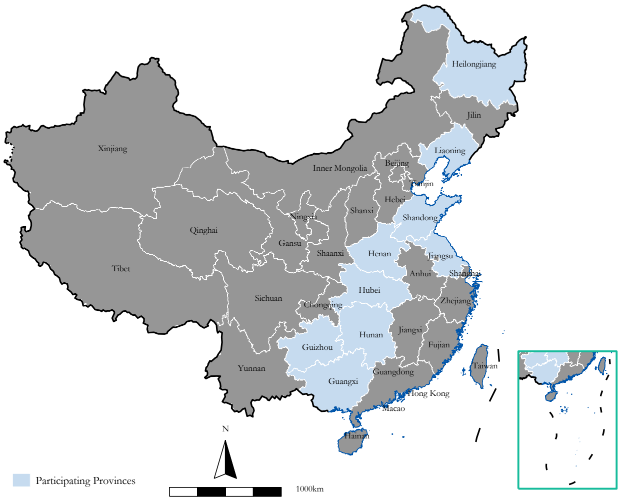

Fig. S9. Provinces Participating in the CHNS between 2001 and 2011. Notes: Data come from the China Health and Nutrition Survey (CHNS).

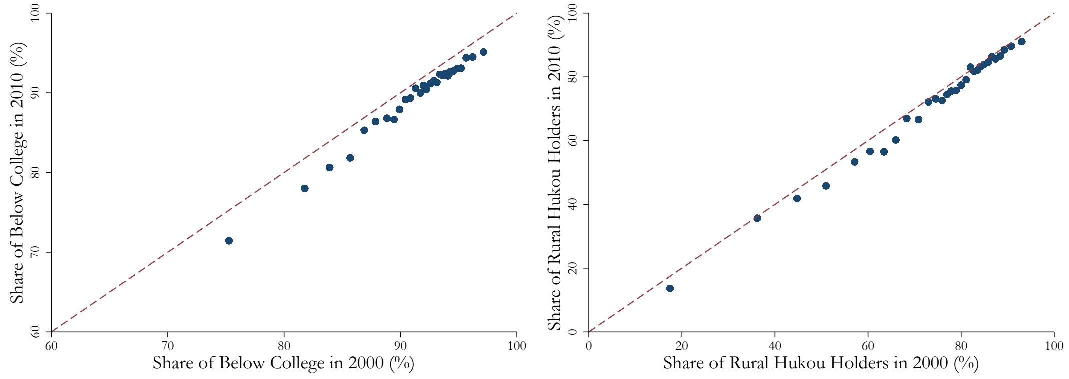

Fig. S10. Persistence of City-level Demographic Characteristics during 2000-2010. Notes: This figure plots measure of SES in 2010 against their values in 2000 for the shares of people without college education (left panel) and the share of rural *hukou* holders (right panel). We divide cites into 30 groups based on the quantile of the share of low-SES individuals in the population in 2000. The x-axis denotes the mean value of the share of low-SES individuals in each quantile in 2000, and the y-axis denotes the mean value of the share of low-SES individuals in each quantile in 2010. Data come from the China Population Censuses.

Table S1. Access to Tap Water by Individual Sociodemographic Characteristics

Panel A: Share with Tap Water Access in 2004				
Demographic Characteristic	Advantaged Group	Disadvantaged Group	Gap in 2004	P-value of Gap in 2004
Below High Scholl	0.708	0.448	0.260	0.000
Below College	0.789	0.478	0.311	0.000
Rural *Hukou* Holders	0.830	0.366	0.463	0.000
Skilled Occupation Workers	0.758	0.399	0.359	0.000
**Panel B: Share with Tap Water Access in 2009**				
Demographic Characteristic	Advantaged Group	Disadvantaged Group	Gap in 2009	P-value of Gap in 2009
Below High Scholl	0.774	0.517	0.257	0.000
Below College	0.810	0.547	0.263	0.000
Rural *Hukou* Holders	0.900	0.433	0.467	0.000
Skilled Occupation Workers	0.803	0.442	0.360	0.000

Notes: This table presents the average share of individuals with access to tap water for advantaged groups (column 2) versus disadvantaged groups (column 3). We present tap water share in the year 2004 (panel A) and 2009 (panel B). We divide individuals into advantaged and disadvantaged groups based on individual-level sociodemographic characteristics (listed in column 1). The difference in tap water access between the two groups of individuals are shown in column 4 and the p-value for mean difference being zero is presented in column 5. Data come from China Health and Nutrition Survey (CHNS).

Table S2. COD Reduction Mandates and Firm-level COD Emissions

	(1)	(2)	(3)
Dep. Var.:	Firm-level COD Emission		
**Panel A : Baseline Results**			
$\lvert$COD Abatement Targets$\rvert$ × Post$_{05}$	-0.206***	-0.165*	-0.190**
	(0.058)	(0.082)	(0.071)
Observations	529,671	529,671	529,671
Firm FE	X	X	X
Year FE	X		
Region X Year FE		X	
Province X Year FE			X
**Panel B : Control for Industry by Year FE**			
$\lvert$COD Abatement Targets$\rvert$ × Post$_{05}$	-0.200***	-0.155*	-0.196**
	(0.055)	(0.079)	(0.076)
Observations	522,729	522,729	522,729
Firm FE	X	X	X
Industry X Year FE	X	X	X
Region X Year FE		X	
Province X Year FE			X

Notes: We leverage COD abatement targets in abosulte value. Two-way robust standard errors clustered at province and year levels are reported in parentheses. *** p<0.01, ** p<0.05, * p<0.1.

Table S3. COD Abatement Targets by Demographic Characteristics

Demographic Indicators	Group Mean		Difference
(Baseline Year in 2000)	<Median	>Median	(1,000 tons)
Share of Below High School	-5.850	-2.421	-3.429***
	(0.559)	(0.197)	(0.593)
Share of Below College	-5.433	-2.837	-2.596***
	(0.544)	(0.265)	(0.605)
Share of Rural *Hukou* Holders	-5.467	-2.803	-2.664***
	(0.543)	(0.263)	(0.604)
Share of Skilled Occupation	-2.831	-5.439	2.607***
	(0.242)	(0.554)	(0.605)

Notes: This table presents the mean COD abatement targets by whether a city has higher or lower share of a particular demographic characteristic (based on the median value) in the baseline year of 2000. Column 2 presents the mean COD abatement targets for cities with population shares of a particular demographic characteristic below the national median, while column 3 reports the corresponding mean for cities above the median. The variable *skilled occupation* refers to individuals employed as public sector staff, professionals, or technicians. Standard error are reported in parentheses. *** $p<0.01$, ** $p<0.05$, * $p<0.1$.

Table S4. Gaps in COD Emissions

	Pre-treatment 2001-2005	Post-treatment 2006-2010	Difference (1,000 tons)
Higher-SES Cities	14.275	12.332	-1.943**
	(0.666)	(0.485)	(0.824)
Lower-SES Cities	12.216	11.384	-0.832
	(0.492)	(0.444)	(0.663)
Difference	2.059**	0.948	
	(0.834)	(0.659)	

Notes: We divide cities into two groups based on the median share of individuals without a college degree in the baseline year of 2000. Data come from the Environmental Survey and Reporting (ESR) and China Population Census. Standard error are reported in parentheses. *** p<0.01, ** p<0.05, * p<0.1.

Table S5. Emission Behaviors at Extensive and Intensive Margins

	(1)	(2)	(3)	(4)
Dep. Var.:	COD Emission (=1)	COD Emission	log(COD Emission)	log(COD Emission/Output)
Sample:	Full Sample	COD Emission >0		
**Panel A: Population Share without College Education**				
Share of Below College × $Post_{05}$	0.004**	0.310**	0.023**	0.015*
	(0.002)	(0.110)	(0.008)	(0.007)
Observations	529,671	481,671	481,671	465,735
**Panel B: Population Share without High School Education**				
Share of Below HS × $Post_{05}$	0.002**	0.207**	0.014**	0.009*
	(0.001)	(0.084)	(0.005)	(0.004)
Observations	529,671	481,671	481,671	465,735
Firm FE	X	X	X	X
Province X Year FE	X	X	X	X

Notes: Two-way robust standard errors clustered at province and year levels are reported in parentheses. *** p<0.01, ** p<0.05, * p<0.1.

Table S6. The Effect on SO_2 Emissions

	(1)	(2)	(3)	(4)
	Firm-level		City-level	
Dep. Var.:	SO2 Emission		SO2 Firm Entry	
Share of Below College × $Post_{05}$	0.570		0.163	
	(0.400)		(0.185)	
Share of Below HS × $Post_{05}$		0.396		0.124
		(0.268)		(0.125)
Observations	500,587	500,587	2,931	2,931
City FE			X	X
Firm FE	X	X		
Two Control Zone X Year FE	X	X	X	X
Province X Year FE	X	X	X	X

Notes: Two-way robust standard errors clustered at province and year levels are reported in parentheses. *** $p<0.01$, ** $p<0.05$, * $p<0.1$.

Table S7. Account for the Effects of Other Water Pollution Policies

	(1)	(2)	(3)	(4)
	City-level	Firm-level	City-level	
Dep. Var.:	COD Emission	COD Emission	Firm Entry	Firm Exit
**Panel A: Control for the Release of the Water Pollution Prevention and Control Plan**				
Share of Below College × $Post_{05}$	0.292**	0.353***	0.609**	0.269
	(0.119)	(0.103)	(0.260)	(0.492)
Observations	3,327	529,671	3,000	2,998
**Panel B: Control for the River Chief Policy**				
Share of Below College × $Post_{05}$	0.291**	0.356***	0.607**	0.283
	(0.119)	(0.106)	(0.257)	(0.485)
Observations	3,327	529,671	3,000	2,998
**Panel C: Control for the Release of the List of National Key Monitoring Firms**				
Share of Below College × $Post_{05}$	0.265*	0.395***	0.583**	0.288
	(0.118)	(0.099)	(0.249)	(0.484)
Observations	3,327	529,671	3,000	2,998
**Panel D: Drop Samples Covered by the Water Pollution Prevention and Control Plan**				
Share of Below College × $Post_{05}$	0.271*	0.295***	0.552*	0.254
	(0.137)	(0.087)	(0.253)	(0.527)
Observations	2,967	469,142	2,760	2,637
**Panel E: Drop Samples Covered by the River Chief Policy**				
Share of Below College × $Post_{05}$	0.290**	0.337***	0.609**	0.302
	(0.119)	(0.100)	(0.258)	(0.490)
Observations	3,317	520,632	2,996	2,988
City FE	X		X	X
Firm FE		X		
Province X Year FE	X	X	X	X

Notes: Two-way robust standard errors clustered at province and year levels are reported in parentheses. *** p<0.01, ** p<0.05, * p<0.1.

Table S8. Baseline Socioeconomic Status and City-level Indicators of Economic Development and Urbanization

	(1)	(2)	(3)	(4)	(5)
Dep. Var.:	Log(GDP)	GDP Growth	Urbanization Rate	Log(Unemployment Rate)	Log(Unemployed Workers)
**Panel A: Population Share without College Education**					
Share of Below College × Post_{05}	0.005	0.096	0.006	-0.000	-0.005
	(0.010)	(0.078)	(0.019)	(0.003)	(0.004)
**Panel B: Population Share without High School Education**					
Share of Below HS × Post_{05}	0.003	0.065	0.008	-0.001	-0.004
	(0.006)	(0.057)	(0.012)	(0.002)	(0.002)
Observations	2,801	2,527	2,831	2,833	2,833
City FE	X	X	X	X	X
Province X Year FE	X	X	X	X	X

Notes: Two-way robust standard errors clustered at province and year levels are reported in parentheses. *** p<0.01, ** p<0.05, * p<0.1.

Table S9. COD Abatement Targets and Changes in City-level Economic and Urbanization Indicators (2005-2010)

	(1)	(2)	(3)	(4)	(5)
Dep. Var.:	Δ GDP	Δ GDP Growth	Δ Urbanization Rate	Δ Unemployment Rate	Δ Unemployed Workers
|COD Abatement Targets|	0.975***	-0.066	0.006	1.088	844.311
	(0.185)	(0.041)	(0.007)	(0.930)	(738.037)
Observations	285	285	283	286	286
Province FE	X	X	X	X	X

Notes: We leverage COD abatement targets in absolute value. Robust standard errors clustered at the province level are reported in parentheses. *** p<0.01, ** p<0.05, * p<0.1.

Table S10. Alternative SES Measures: Rural *Hukou* Holders and Skilled Occupation

	(1)	(2)	(3)	(4)
	City-level	Firm-level	City-level	
Dep. Var.:	COD Emission	COD Emission	Firm Entry	Firm Exit
**Panel A: Share of Rural Hukou Holders**				
Share of Rural *Hukou* Holders × Post_{05}	0.077**	0.086**	0.190*	0.003
	(0.026)	(0.032)	(0.094)	(0.137)
**Panel B: Share of Skilled Occupation**				
Share of Skilled Occupation × Post_{05}	-0.341**	-0.535*	-0.890*	-0.728
	(0.116)	(0.267)	(0.442)	(0.830)
Observations	3,327	529,671	3,000	2,998
City FE	X		X	X
Firm FE		X		
Province X Year FE	X	X	X	X

Notes: Two-way robust standard errors clustered at province and year levels are reported in parentheses. *** p<0.01, ** p<0.05, * p<0.1.

Table S11. Heterogeneous Effects by Baseline Income and Minority Shares

	(1)	(2)	(3)	(4)
	City-level	Firm-level	City-level	
Dep. Var.:	COD Emission	COD Emission	Firm Entry	Firm Exit
**Panel A: Effects by Baseline Average Income in 2000**				
Log(Income) × $Post_{05}$	-6.554*	-7.791**	-29.572**	18.064
	(3.504)	(2.934)	(10.505)	(16.019)
Observations	2,630	499,952	2,367	2,367
**Panel B: Effects by Baseline Minority Shares in 2000**				
Share of Minorities × $Post_{05}$	0.048**	0.176	0.066*	-0.146***
	(0.021)	(0.115)	(0.031)	(0.041)
Observations	3,327	529,671	3,000	2,998
City FE	X		X	X
Firm FE		X		
Province X Year FE	X	X	X	X

Notes: Two-way robust standard errors clustered at province and year levels are reported in parentheses. *** p<0.01, ** p<0.05, * p<0.1.

Table S12. Control for Industry by Year FE

	(1)	(2)	(3)
Dep. Var.:		Firm-level COD Emission	
**Panel A: Population Share without College Education**			
Share of Below College × Post_{05}	0.358***	0.327**	0.400***
	(0.106)	(0.128)	(0.121)
**Panel B: Population Share without High School Education**			
Share of Below HS × Post_{05}	0.232**	0.226**	0.293**
	(0.079)	(0.090)	(0.092)
Observations	529,671	522,729	513,004
Firm FE	X	X	X
Province X Year FE	X	X	
Industry X Year FE		X	
Region X Industry X Year FE			X

Notes: We categorize regions as north, central, east, south, southwest, northwest, and northeast China. Two-way robust standard errors clustered at province and year levels are reported in parentheses. *** p<0.01, ** p<0.05, * p<0.1.

Table S13. Robustness of Emission Actives: Multiple Hypothesis Testing

Panel A: City-level Regression			
	(1)	(2)	(3)
Dep. Var.:	COD Emission	Firm Entry	Firm Exit
Share of Below College × $Post_{05}$	0.292***	0.623***	0.280
Romano and Wolf P-value	[0.000]	[0.000]	[0.390]
P-value of Baseline Estimates	[0.037]	[0.047]	[0.582]
Observations	3,327	3,000	2,998
City FE	X	X	X
Province X Year FE	X	X	X

Panel B: Firm-level Regression					
	(1)	(2)	(3)	(4)	(5)
Dep. Var.:	COD Emission	COD Emission(=1)	COD Emission	log(COD Emission)	ln(COD Emission/Output)
Sample:	Full Sample		COD Emission >0		
Share of Below College × $Post_{05}$	0.358***	0.004***	0.310***	0.023***	0.015***
Romano and Wolf P-value	[0.000]	[0.000]	[0.000]	[0.000]	[0.000]
P-value of Baseline Estimates	[0.008]	[0.024]	[0.020]	[0.022]	[0.075]
Observations	529,671	529,671	481,671	481,671	465,735
Firm FE	X	X	X	X	X
Province X Year FE	X	X	X	X	X

Notes: We use the approach proposed by Romano and Wolf (1, 2) and estimate step-down adjusted p-values robust to multiple hypothesis testing. *** p<0.01, ** p<0.05, * p<0.1.

Table S14. The Power of Pretrend Tests (for Fig. 2 and *SI Appendix*, Fig. S3)

Estimated Treament Effects	Power of Rejection	Smallest Slope of Pretrend in Absolute Value
**Panel A: Share of Below College**		
0.358	80%	0.041
	90%	0.048
**Panel B: Share of Below HS**		
0.232	80%	0.041
	90%	0.048
**Panel C: Share of Rural *Hukou* Holders**		
0.086	80%	0.025
	90%	0.029
**Panel D: Share of Skilled Occupation**		
-0.535	80%	0.138
	90%	0.162

Table S15. Difference-in-Differences Estimates with a Binary Treatment Variable

	(1)	(2)	(3)	(4)
Dep. Var.:	City Level-COD Emission		Firm Level-COD Emission	
Above-median Dummy X Post	2.408**		3.174**	
	(0.990)		(1.173)	
Above-mean Dummy X Post		2.508**		3.737**
		(1.015)		(1.634)
Observations	3,327	3,327	529,671	529,671
City FE	X	X		
Firm FE			X	X
Province X Year FE	X	X	X	X

Notes: We perform difference-in-differences regressions with a binary treatment variable. In columns 1 and 2, the treatment variable, i.e., the above-median dummy, is defined based on whether the share of below-college population is above the median level. In columns 3 and 4, the treatment variable, i.e., the above-mean dummy, is defined based on whether the share of below-college population is above the average level. Two-way robust standard errors clustered at province and year levels are reported in parentheses. *** p<0.01, ** p<0.05, * p<0.1.

Table S16. Weighted Average Slope (WAS) Estimates: Two-Period Panel for 2005 and 2010

	(1)	(2)	(3)	(4)
Dep. Var.:	City Level-COD Emission		Firm Level-COD Emission	
Share of Below College × Post_{05}	0.278**		1.093***	
	(0.140)		(0.237)	
Share of Below HS × Post_{05}		0.251***		0.973***
		(0.084)		(0.171)
City FE	X	X		
Firm FE			X	X
Province X Year FE	X	X	X	X

Notes: We limit our sample to the years 2005 and 2010. This table presents the WAS estimates of the coefficients on the interaction between the share of low-SES population (i.e. share of individuals without a high school or a college education) and the post-2005 dummy. *** p<0.01, ** p<0.05, * p<0.1.

Table S17. Control for Other Concurrent Water Pollution Control Policies

	(1)	(2)	(3)	(4)	(5)
Dep. Var.:	Cardiovascular Disease	Inactive Days	Tumor	Daily Work Hours	Wage Percentile
**Panel A: Control for the Release of the Water Pollution Prevention and Control Plan**					
Below College × NoTap × $Post_{05}$	0.043**	0.271	0.008*	-0.704**	-10.037**
	(0.014)	(0.148)	(0.003)	(0.224)	(2.552)
**Panel B: Control for the River Chief Policy**					
Below College × NoTap × $Post_{05}$	0.043**	0.273	0.008*	-0.717**	-10.035**
	(0.014)	(0.150)	(0.003)	(0.226)	(2.504)
**Panel C: Control for the Release of the List of National Key Monitoring Firms**					
Below College × NoTap × $Post_{05}$	0.044**	0.268	0.008*	-0.728**	-10.070**
	(0.014)	(0.148)	(0.003)	(0.220)	(2.654)
Observations	25,097	25,097	28,936	4,801	3,337
Individual FE	X	X	X	X	X
Province X Year FE	X	X	X	X	X

Notes: Individual level regressions. We drop observations with missing values in dependent variables, tap water access and education. Two-way robust standard errors clustered at province and year levels are reported in parentheses. *** p<0.01, ** p<0.05, * p<0.1.

Table S18. Control for Access to Health Facilities and Age Effects

	(1)	(2)	(3)	(4)	(5)
Dep. Var.:	Cardiovascular Disease	Inactive Days	Tumor	Daily Work Hours	Wage Percentile
**Panel A: Control for Access to Health Facilities**					
Below College × NoTap × Post_{05}	0.046**	0.273	0.008*	-0.617*	-10.822**
	(0.013)	(0.153)	(0.003)	(0.260)	(3.877)
Observations	25,097	25,097	28,936	4,801	3,337
Individual FE	X	X	X	X	X
Province X Year FE	X	X	X	X	X
**Panel B: Control for Age Effects**					
Below College × NoTap × Post_{05}	0.054**	0.286*	0.006	-0.675**	-8.995**
	(0.019)	(0.117)	(0.003)	(0.215)	(2.492)
Observations	25,087	25,087	28,926	4,792	3,317
Age X Below College FE	X	X	X	X	X
Age X NoTap FE	X	X	X	X	X
Individual FE	X	X	X	X	X
Province X Year FE	X	X	X	X	X

Notes: Individual level regressions. We drop observations with missing values in dependent variables, tap water access and education. Two-way robust standard errors clustered at province and year levels are reported in parentheses. *** p<0.01, ** p<0.05, * p<0.1.

Table S19. Placebo Tests

	(1)	(2)	(3)	(4)
Dep. Var.:	Cardiovascular Disease	Inactive Days	Tumor	Health Index
**Panel A: Access to Indoor Bathroom**				
Below College × NoBathroom × $Post_{05}$	0.007	0.021	0.006	0.046
	(0.016)	(0.122)	(0.005)	(0.072)
Observations	25,532	25,532	29,460	25,390
**Panel B: Randomize Access to Tap Water**				
Below College × NoTap × $Post_{05}$	-0.023	0.037	0.001	-0.009
	(0.018)	(0.112)	(0.006)	(0.071)
Observations	25,097	25,097	28,936	24,965
**Panel C: Randomize Education Level**				
Below College × NoTap × $Post_{05}$	-0.001	0.058	0.000	0.047
	(0.010)	(0.064)	(0.002)	(0.049)
Observations	25,097	25,097	28,936	24,965
Individual FE	X	X	X	X
Province X Year FE	X	X	X	X

Notes: Individual level regressions. We drop observations with missing values in dependent variables, tap water access and education. In column 4, we use an overall health problem index as the dependent variable. Specifically, we construct an inverse-covariance weighted summary index of different health outcomes: the incidence of tumor and cardiovascular disease, and the number of days that an individual cannot perform his or her routine daily activities due to illnesses. Two-way robust standard errors clustered at province and year levels are reported in parentheses. *** p<0.01, ** p<0.05, * p<0.1.

Table S20. Impact on Other Diseases

Dep. Var.:	(1)<br>Asthma	(2)<br>Respiratory Disease	(3)<br>Muscle Aches Pains	(4)<br>ENT Disease
Below College × NoTap × $Post_{05}$	0.002	-0.015	-0.012	0.005
	(0.003)	(0.023)	(0.010)	(0.013)
Below College × $Post_{05}$	0.001	0.012	0.002	0.001
	(0.002)	(0.011)	(0.007)	(0.009)
Observations	29,079	19,194	18,572	18,448
Individual FE	X	X	X	X
Province X Year FE	X	X	X	X

Notes: Individual level regressions. We drop observations with missing values in dependent variables, tap water access and education. Two-way robust standard errors clustered at province and year levels are reported in parentheses. *** p<0.01, ** p<0.05, * p<0.1.

Table S21. Control for Air Pollution

	(1)	(2)	(3)	(4)	(5)
Dep. Var.:	Cardiovascular Disease	Inactive Days	Tumor	Daily Work Hours	Wage Percentile
Below College × NoTap × $Post_{05}$	0.043*	0.273	0.008*	-0.744*	-10.175**
	(0.017)	(0.140)	(0.003)	(0.268)	(2.584)
Below College × $Post_{05}$	0.004	-0.032	-0.003	-0.033	-0.932
	(0.018)	(0.067)	(0.002)	(0.122)	(2.997)
Observations	25,097	25,097	28,936	4,801	3,337
Individual FE	X	X	X	X	X
Province X Year FE	X	X	X	X	X

Notes: Individual level regressions. We drop observations with missing values in dependent variables, tap water access and education. Two-way robust standard errors clustered at province and year levels are reported in parentheses. *** $p<0.01$, ** $p<0.05$, * $p<0.1$.

Table S22. Robustness of Health and Labor Outcomes: Multiple Hypothesis Testing

	(1)	(2)	(3)	(4)	(5)
Dep. Var.:	Cardiovascular Disease	Inactive Days	Tumor	Daily Work Hours	Wage Percentile
Below College × NoTap × Post_{05}	0.044*	0.270	0.008*	-0.727*	-10.035*
Romano and Wolf P-value	[0.070]	[0.100]	[0.070]	[0.070]	[0.050]
P-value of Baseline Estimates	[0.036]	[0.142]	[0.055]	[0.031]	[0.016]
Observations	25,097	25,097	28,936	4,801	3,337
Individual FE	X	X	X	X	X
Province X Year FE	X	X	X	X	X

Notes: We use the approach proposed by Romano and Wolf (1, 2) and estimate step-down adjusted p-values robust to multiple hypothesis testing. *** p<0.01, ** p<0.05, * p<0.1.

Table S23. Changes in City-level Tap Water Coverage, 2005-2010 (Census Data)

	(1)	(2)	(3)	(4)
Dep. Var.:	Changes in Tap Water Coverage Rates from 2005 to 2010			
Share of Below HS	0.150			
	(0.144)			
Share of Below College		0.241		
		(0.200)		
Share of Rural *Hukou* Holders			0.072	
			(0.063)	
Share of Skilled Occupation				-0.476
				(0.371)
|COD Abatement Targets|	-0.123	-0.135	-0.146	-0.134
	(0.152)	(0.148)	(0.149)	(0.146)
Observations	337	337	337	337
R-squared	0.015	0.015	0.016	0.017

Notes: We regress changes in city-level tap water coverage rates (i.e., the share of the population with access to tap water) between 2005 and 2010 on the population share of low-SES individuals at baseline and city-specific COD reduction targets (in absolute value). We calculate city-level tap water coverage rates using data from the 2005 and 2010 Population Censuses. Robust standard errors clustered at the province level are reported in parentheses. *** $p<0.01$, ** $p<0.05$, * $p<0.1$.

Table S24. Community-level Skill-specific Tap Water Coverage (CHNS)

	(1)	(2)	(3)
Dep. Var.:	Community-level Skill-specific Tap Water Coverage Rates		
Below College Group (=1) × Post_{05}	0.023	0.023	0.025
	(0.026)	(0.026)	(0.023)
Below College Group (=1)	-0.131***	-0.130***	-0.123***
	(0.023)	(0.023)	(0.021)
Post_{05}	0.009		
	(0.014)		
Observations	1,475	1,475	1,469
City FE	X	X	
Year FE		X	
City X Year FE			X

Notes: Using five waves of CHNS data (2000, 2004, 2006, 2009, and 2011), we construct community- and year-level tap water coverage rates (i.e., the share of individuals with access to tap water) separately for those with a college degree and those without. Some communities include both college graduates and individuals without a college degree. In such cases, we calculate the share of college-educated individuals with access to tap water in a given community, as well as the share of non-college-educated individuals with access to tap water in that community. We then regress the community-level skill-specific tap water coverage rates on the interaction between the below college group indicator and the post-2005 indicator. Two-way robust standard errors clustered at province and year levels are reported in parentheses. *** p<0.01, ** p<0.05, * p<0.1.

Table S25. Baseline Tap Water Access within Cities Does Not Predict Changes in Emissions

	(1)	(2)	(3)	(4)
Dep. Var.:	Δ 5-Year Average COD (2001-05 vs. 2006-10)	ΔCOD2006-10	ΔCOD 2001-10	ΔCOD 2001-2005
Access to Tap Water (%)	-2.496	-3.045	-4.184	0.463
	(2.251)	(2.548)	(3.612)	(3.520)
Observations	2,332	2,332	2,332	2,332
City FE	YES	YES	YES	YES

Notes: The geographic unit of our analysis is the prefecture-level city, which typically consists of about 10 counties.We regress county-level changes in COD emissions on the baseline share of individuals with tap water access in each county in 2000 and control for city fixed effects. The dependent variables are the county-level changes in five-year average COD emissions before and after the enactment of the policy (column 1) and the county-level changes in annual COD emissions between 2006 and 2010 (column 2), between 2001 and 2010 (column 3), and between 2001 and 2005 (column 4). Robust standard errors clustered at the province level are reported in parentheses. *** p<0.01, ** p<0.05, * p<0.1.

Table S26. Differential Sorting Responses to China's 11 FYP by Group

	(1)	(2)	(3)	(4)
Dep. Var.:	Move Indicator			
SES Group:	Below College		Below High School	
Group Indicator $\times$ Post$_{05}$	0.000279	0.000927	-0.000382	-0.000254
	(0.00162)	(0.00184)	(0.000677)	(0.000684)
Observations	216,090	236,561	216,090	236,561
Individual FE	X	X	X	X
Region X Year FE	X		X	
Province X Year FE		X		X

Notes: Individual level regressions. We employ individual longitudinal panel data, constructed using the China Labor-force Dynamic Survey (an alternative data source), and track individuals from 1999 to 2016. The dependent variable is indicator for whether individual i change his/her city location between year t and year $t-1$. Two-way robust standard errors clustered at province and year levels are reported in parentheses. *** p<0.01, ** p<0.05, * p<0.1.

Table S27. Missing Value

	(1)	(2)	(3)
Dep. Var.:	Missing Cardiovascular Disease (=1)	Missing Inactive Days (=1)	Missing Labor Outcome (=1)
Below College × NoTap × Post_{05}	0.013	0.012	-0.045
	(0.022)	(0.013)	(0.051)
Observations	33,101	33,101	33,101

Notes: The table presents the triple differences specification that uses an indicator for whether cardiovascular disease, inactive days, or labor outcome (daily work hours, wage) is missing for a particular respondent as the dependent variable. There are no missing values for the incidence of tumor. Robust standard errors clustered at the individual level are reported in parentheses. *** p<0.01, ** p<0.05, * p<0.1.

Table S28. Descriptive Statistics

Variable	Mean	Std. Dev.
**Panel A: Baseline City Demographic Characteristics in 2000**		
Share of Below High School (%)	81.644	7.699
Share of Below College (%)	91.021	4.746
Share of Rural Hukou Holders (%)	72.719	17.427
Share of Skilled Occupation Workers (%)	7.227	2.817
**Panel B: City- and Firm- level Pollution Measures**		
City-level COD Emissions (1,000 tons)	12.568	15.349
Firm-level COD Emissions (ton)	33.263	105.939
City-level Firm Entry	17.195	25.730
City-level Firm Exit	36.741	68.560
**Panel C: Individual Health and Labor Outcomes**		
Cardiovascular Disease(=1)	0.111	0.314
Inactive Days due to Illness	0.160	1.238
Tumor(=1)	0.004	0.066
Daily Work Hours	7.823	1.850
Wage Percentile	58.024	27.784

Table S29. Robustness of Emission Actives: Bootstrapped Errors

	(1)	(2)	(3)	(4)
	City-level	Firm-level	City-level	
Dep. Var.:	COD Emission (1,000 tons)	COD Emission (ton)	Firm Entry	Firm Exit
**Panel A: Population Share without College Education**				
Share of Below College × $Post_{05}$	0.292**	0.358**	0.623**	0.280
P-value	[0.026]	[0.020]	[0.046]	[0.622]
95% Confidence Interval	[0.055, 0.580]	[0.085, 0.620]	[0.013, 1.269]	[-0.846, 1.362]
**Panel B: Population Share without High School Education**				
Share of Below HS × $Post_{05}$	0.218**	0.232**	0.453*	0.089
P-value	[0.028]	[0.026]	[0.051]	[0.816]
95% Confidence Interval	[0.043, 0.433]	[0.031, 0.423]	[-0.006, 0.933]	[-0.680, 0.827]
Observations	3,327	529,671	3,000	2,998
City FE	X		X	X
Firm FE		X		
Province X Year FE	X	X	X	X

Notes: We report wild bootstrap cluster p-values and 95% confidence interval in square brackets. *** p<0.01, ** p<0.05, * p<0.1.

Table S30. Robustness of Health and Labor Outcomes: Bootstrapped Errors

	(1)	(2)	(3)	(4)	(5)
Dep. Var.:	Cardiovascular Disease	Inactive Days	Tumor	Daily Work Hours	Wage Percentile
Below College × NoTap × $Post_{05}$	0.044**	0.270*	0.008*	-0.727*	-10.035**
P-value	[0.050]	[0.074]	[0.054]	[0.054]	[0.021]
95% Confidence Interval	[0.002, 0.086]	[-0.058, 0.711]	[-0.000, 0.017]	[-1.388, -0.033]	[-19.450, -2.897]
Observations	25,097	25,097	28,936	4,801	3,337
Individual FE	X	X	X	X	X
Province X Year FE	X	X	X	X	X

Notes: We report wild bootstrap cluster p-values and 95% confidence interval in square brackets. *** p<0.01, ** p<0.05, * p<0.1.

Supporting Information Text

Data Appendix

Firm-level emissions data. Firm-level emissions data come from the Environmental Survey and Reporting (ESR) database, which is a large administrative dataset collected by the Ministry of Environmental Protection (MEP) of China (or the former State Environmental Protection Administration). The MEP began to conduct this nationwide survey in the early 1980s to gather detailed information on environmental pollutants and wastes.

Since 2001, the scope of the ESR has been determined by the pollution discharges of individual firms/plants and the total amount of discharge at the county level. The ESR covers all top-emitting firms/plants contributing 85% of total emissions of the major pollutants in a county. In particular, these major pollutants include wastewater, chemical oxygen demand, ammonia nitrogen, sulfur dioxide, nitrogen oxides, smoke, dust, and industrial solid waste. Consequently, the ESR contains all local major emission sources in China, like heavily polluting industrial firms, urban sewage treatment plants, and hospitals. These key emitters are required to report detailed information on major pollutant emissions along with other firm attributes. For firms with complex administrative structures, such as joint enterprises, their branch plants are treated as the basic units for reporting pollution information.

In this paper, we use data recorded in the ESR database. Specifically, the MEP requires key polluting enterprises to complete a standardized reporting form titled *Pollutant Emissions and Treatment Status of Industrial Enterprises*. This form collects information on firm name, enterprise legal entity code, address, industry classification code, year of commencement of operation, output, and emissions of major pollutants. The relevant survey questions employed in this study are listed below. Additional details regarding the complete structure and content of the original reporting form are available on the official website of the MEP (https://www.mee.gov.cn/gkml/zj/wj/200910/W020070917492947218703.pdf). Access to firm-level emissions data is restricted; however, researchers may apply for the data for research purposes from certain Chinese universities or institutions or through the MEP.

Key Survey Questions Recorded in ESR Database:

1.Year of Completion: □ □ □ □

2.Enterprise Legal Entity Code: □ □ □ □ □ □ □ □ □ – □ (□ □)

3.Firm Name: ____________________

4.Address and Administrative Division Code: ____________________ (□ □ □ □ □ □)

5.Industry Classification: ____________________ (□ □ □ □)

6.Year of Commencement of Operation: □ □ □ □

7.Pollutant Emissions and Treatment Status:

Code	Indicator Name	Unit	This Year Actual
I	I. Basic Information of the Enterprise	–	–
	I.1. Total Industrial Output Value (Current Price)	10,000 Yuan	
II	II. Industrial Wastewater	–	–
	II.10. Discharge Amount of Pollutants in Industrial Wastewater	–	–
	II.10.(1) Discharge Amount of COD	kg	

Following the literature on pollution in China, we use Chemical Oxygen Demand (COD) emissions as a measure of firm-level water pollution. Our analysis is based on longitudinal firm-level emissions data from the ESR, covering the period from 2001 to 2010. We clean the sample by excluding firms with missing address information and those without any reported COD emissions throughout the sample period. The resulting baseline panel dataset tracks 127,375 water-polluting enterprises over this time frame. We further compute the number of polluting firm entrants in each city using the reported year of commencement of operation from 2001 to 2009 (the information about the year of commencement of operation is missing in 2010.). We also calculate the number of polluting firm exits in each year at the city level.

Population Census Data. The Fifth National Census of China was conducted by the State Council of the People's Republic of China in 2000. This census aimed to gather comprehensive demographic data about the population of China, including population size, demographic distribution, and various socio-economic factors. Each province, autonomous region, and municipality conducted a survey by randomly sampling 10% of households and collective units within each investigation area to gather data using long-form census questionnaires. This sampling approach was used to ensure a representative selection of the population of subnational regions within China. *

The census data contain a wide range of variables about demographic and economic characteristics of individuals, such as residential address, age, gender, educational attainment, *hukou* type (rural/urban), and occupation. The census survey questions relevant to the construction of key variables in this study are presented below. The city-level census data, along with the questionnaire, are accessible at the National Bureau of Statistics (https://www.stats.gov.cn/sj/pcsj/rkpc/5rp/index.htm). While city-level census data are publicly available, individual-level data are restricted access. Researcher may apply for access to individual-level census data from the National Bureau of Statistics or through certain Chinese universities or institutions.

Key Survey Questions Recorded in Population Census Data:

Address and Administrative Division Code: ______(Province) ______(City) ______(County)

R3. Gender:
- 1 = Male
- 2 = Female

R4. Age: __________________ (Birth year: ☐ ☐ ☐ ☐)

R5. Ethnic Group: __________________

R6. Household Registration Status
- 1 = Registered with *Hukou* in this township/street and currently living here.
- 2 = Registered with *Hukou* elsewhere but living in this township/street for six months or more
- 3 = Registered with *Hukou* elsewhere but living in this township/street for less than six months
- 4 = *Hukou* registration status pending
- 5 = Temporarily without *hukou*

R7. *Hukou* Type:
- 1 = Agricultural (rural)
- 2 = Non-agricultural (urban)

R15. Educational Attainment:
- 1 = No schooling
- 2 = Literacy class
- 3 = Primary school
- 4 = Middle school
- 5 = High school
- 6 = Technical or vocational degree
- 7 = College degree
- 8 = University/Bachelor's degree
- 9 = Graduate degree

R20. Occupation: __________________ (Occupation classification code: ☐ ☐ ☐)

H18: With access to tap water?
- 1 = Yes
- 2 = No

We use these data to categorize a city's baseline social and economic disadvantage in the main analysis of firm behaviors. In particular, we use individual-level data to calculate the share of the population without a high school degree (corresponding to categories 1 to 4 in question R15 on educational attainment), the share of the population without a college degree (corresponding to categories 1 to 5 in question R15 on educational attainment), the share of rural *hukou* holders in the population (where *hukou* type is classified as agricultural *hukou* in question R7), and the share of the population with a skilled occupation in each city (defined as those with occupation classification codes beginning with 0, 1, or 2 in question R20). †

* The 2000 Population Census includes approximately 130 million individuals, and we use a 9% random subsample of the census data.

† Code 0 refers to public sector staff, while codes 1 and 2 correspond to professionals and technicians.

China Health and Nutrition Survey. The China Health and Nutrition Survey (CHNS) is jointly conducted by the University of North Carolina at Chapel Hill and the Chinese Center for Disease Control and Prevention, and aims to collect detailed microdata on health conditions, nutrition intake, physical activity, behavior changes, and family planning policies and programs (implemented by national and local governments).

The CHNS was designed to provide representation of rural, urban, and suburban areas that vary substantially in geography, economic development, public resources, and health indicators (3). A multistage, random cluster process was used to draw the sample surveyed in each of the provinces. [‡] Since our analysis focuses on the water pollution policy implemented under China's 11th Five-Year Plan (2006–2010), we use five waves of the CHNS from 2000 to 2011 (i.e., 2000, 2004, 2006, 2009, and 2011). Our longitudinal sample covers approximately 3,600 households with over 6,900 individuals across 9 provinces and 49 municipal cities. These provinces include Liaoning, Heilongjiang, Jiangsu, Shandong, Henan, Hubei, Hunan, Guangxi, Guizhou.

The CHNS surveyed respondents to gather their information about lifestyle, access to public infrastructure, demographic attributes, health status, and labor market outcomes. We are particularly interested in the use of clean tap water, health status, and economic status. Below, we provide the related survey questions. The survey data, along with the questionnaire, are accessible at the China Health and Nutrition Survey Website (https://www.cpc.unc.edu/projects/china).

Key Survey Questions Recorded in CHNS:

T1. Province:
- 21 = Liaoning
- 23 = Heilongjiang
- 32 = Jiangsu
- 37 = Shandong
- 41 = Henan
- 42 = Hubei
- 43 = Hunan
- 45 = Guangxi
- 52 = Guizhou

T2. Urban/Rural Site:
- 1 = Urban site
- 2 = Rural site

T3. City:
- 1 = First city
- 2 = Second city

T5. Household Number: ___________________

L3. What is the source of drinking water?
- 1 = ground water (>5 meters)
- 2 = open well ($\leq$ 5 meters)
- 3 = creek, spring, river, lake
- 4 = ice/snow
- 5 = water plant
- 7 = others
- 9 = unknown

A3a. Age(years): ___________________

*Record 018 if 18.00-18.99 years, 019 if 19.00-19.99 years, etc.

AA2a. Sex:
- 1 = Male
- 2 = Female

A8b1. To which type of household registration do you belong?
- 1 = urban
- 2 = rural

A12. What is the highest level of education you have attained?
- 1 = graduated from primary school
- 2 = lower middle school degree
- 3 = upper middle school degree
- 4 = technical or vocational degree
- 5 = university or college degree
- 6 = master's degree or higher
- 9 = unknown

C6. For how many hours in a day, on the average, did you work? ___________________

[‡] The CHNS typically selects cities and counties stratified by income within each province. Villages and townships within the counties and urban/suburban neighborhoods within the cities are selected randomly.

*If "unknown," record -9.

C8. On the average, what was your monthly wage/salary in last year, including subsidies? (yuan) ___________________

*If "unknown," record -9999.

M26a. For how many days during the past 4 weeks were you unable to carry out normal activities due to this illness? ___________________

*If "unknown," record -9.

U22. Has a doctor ever told you that you suffer from high blood pressure?

0 = no (skip to Question 4)

1 = yes

9 = unknown (skip to Question 4)

U24j. Has a doctor ever given you the diagnosis of myocardial infarction?

0 = no (skip to Question 9)

1 = yes

9 = unknown (skip to Question 9)

U24l. Has a doctor ever given you the diagnosis of stroke or transient ischemic attack?

0 = no (skip to Question 14)

1 = yes

9 = unknown (skip to Question 14)

U24x. How old were you when you were first diagnosed with tumor? (years) ___________________

*If "unknown," record -9.

M52. Did you seek care from a formal medical provider during the past 4 weeks?

0 = no (skip to Question 15)

1 = yes

M40. What was the doctor's diagnosis of your illness or injury?

00 = no diagnosis	12 = eye/ear/nose/throat/teeth disease
01 = infectious/parasitic disease	13 = digestive disease
02 = heart disease	14 = urinary disease
03 = tumor	15 = sexual dysfunction
04 = respiratory disease	16 = obstetrical/gynecological disease
05 = injury	17 = neonatal disease
06 = alcohol poisoning	18 = dermatological disease
07 = endocrine disorder	19 = muscular/rheumatological disease
08 = hematological disease	20 = genetic disease
09 = mental/psychiatric disorder	21 = old age/mid-life syndrome
10 = mental retardation	22 = other (specify:___________________)
11 = neurological disorder	-9 = Unknown

For educational attainment, question A12 asks: "What is the highest level of education you have attained?" We construct a binary indicator for individuals without a college degree (or a technical or vocational degree), which takes the value of one if the respondent selects categories 1 to 3 in wave 2000, and zero otherwise.

For the use of clean tap water, question L3 asks: "What is the source of drinking water for your household? (If more than one source, record the most important one)" The numerals 1 to 9 indicate the following sources. 1 for "ground water (>5 meters)", 2 for "open well (<= 5 meters)", 3 for "creek, spring, river, lake", 4 for "ice/snow", 5 for "water plant", 7 for "others", and 9 for "unknown". We define a binary indicator for households without tap water access, which equals one if the respondent chooses 1 to 4 and zero otherwise. Only 0.7% of households choose 7 and 9; we drop observations with responses of 7 and 9.

For cardiovascular disease, questions U22, U24j, and U24l ask: "Has a doctor ever told you that you suffer from high blood pressure/myocardial infarction/ stroke/transient ischemic attack?". We construct a binary indicator for individuals with cardiovascular disease, which takes the value of one if the respondent selects category 1 for any of these three questions, and zero otherwise. Question M26a also asks: "For how many days during the past 4 weeks were you unable to carry out normal activities due to illness?" For the incidence of tumors, we use responses to question U24x from the 2011 and 2015 CHNS waves and to questions M40 and M52 from the 2000, 2004, 2006, 2009, and 2011 waves. Based on responses to these questions, we obtain information on the first year in which each respondent was diagnosed with a tumor during our sample period (2001–2010).

For labor market outcomes, the questionnaire asks: "For how many hours in a day, on the average, did you work?" and "On the average, what was your monthly wage/salary, including subsidies? (Chinese Yuan)"

We focus on the sample of individuals with information on tap water access and education, and restrict our data to those who appear both before and after China's 11th FYP in the survey. Within this sample, health and illness are surveyed for most individuals, but wage and labor supply information is collected for a random subset sample of smaller size. When analyzing the effects on health outcomes, we drop individuals with missing information on health outcomes. Similarly, when analyzing labor market consequences, we drop individuals with missing information on labor outcomes. We provide evidence showing

that the probability of having missing values (across different dependent variables) is not systematically associated with our main effects of interest. Specifically, we show in *SI Appendix*, Table S27 that the main coefficient in our triple-differences specification is not statistically significant.

References

1. JP Romano, M Wolf, Exact and approximate stepdown methods for multiple hypothesis testing. *J. Am. Stat. Assoc.* **100**, 94–108 (2005).
2. JP Romano, M Wolf, Stepwise multiple testing as formalized data snooping. *Econometrica* **73**, 1237–1282 (2005).
3. B Zhang, F Zhai, S Du, BM Popkin, The China health and nutrition survey, 1989–2011. *Obes. Rev.* **15**, 2–7 (2014).
